# Traumatic Brain Injury Intensive Evaluation and Treatment Program: Protocol for a Partnered Evaluation Initiative Mixed Methods Study

**DOI:** 10.2196/44776

**Published:** 2023-05-09

**Authors:** Jolie N Haun, Risa Nakase-Richardson, Christine Melillo, Jacob Kean, Rachel C Benzinger, Tali Schneider, Mary Jo V Pugh

**Affiliations:** 1 James A. Haley Veterans' Hospital Research Service Tampa, FL United States; 2 College of Public Health University of South Florida Tampa, FL United States; 3 Pulmonary/Sleep Medicine Division Department of Internal Medicine University of South Florida Tampa, FL United States; 4 VA Salt Lake City Health Care System VA Informatics and Computing Infrastructure Salt Lake City, UT United States; 5 VA Salt Lake City Health Care System Informatics, Decision-Enhancement and Analytic Sciences Center Salt Lake City, UT United States

**Keywords:** service member, rehabilitation, traumatic brain injury, TBI, posttraumatic stress disorder, PTSD, pain, military, brain injury, trauma, traumatic, participatory, recovery, veteran, implementation, service delivery, protocol, treatment program, implementation, health care implementation, Consolidated Framework for Implementation Research, CFIR, cognitive, cognition, brain, script, Bayesian, network analysis, directed acyclic graph, effect size, missing data, inpatient, modality

## Abstract

**Background:**

The traumatic brain injury (TBI) Intensive Evaluation and Treatment Program (IETP) is an innovative modality for delivering evidence-based treatments in a residential, inpatient format to special operational forces service members and veterans with mild TBI. IETPs provide bundled evidence-based assessment, treatment, referral, and case management in concordance with the existing guidelines for mild TBI and commonly co-occurring comorbidities. To date, there has been no formal characterization or evaluation of the IETP to understand the determinants of implementation across the system of care. The goal of our partnered evaluation initiative (PEI) with an operational partner, the Physical Medicine and Rehabilitation National Program Office, is to facilitate the full implementation of the IETP across all 5 Veterans Health Administration TBI–Centers of Excellence (TBI-COE) and to inform minimum standards while supporting the unique characteristics of each site.

**Objective:**

This IETP partnered evaluation will describe each of the 5 TBI-COE IETP services and state of implementation to identify opportunities for adaptation and scale, characterize the relationship between patient characteristics and clinical services received, evaluate the outcomes for participants in the IETP, and inform ongoing implementation and knowledge translation efforts to support IETP expansion. In alignment with the goals of the protocol, ineffective treatment components will be targeted for deimplementation.

**Methods:**

A 3-year concurrent mixed methods evaluation using a participatory approach in collaboration with the operational partner and TBI-COE site leadership will be conducted. Qualitative observations, semistructured focus groups, and interviewing methods will be used to describe IETP, stakeholder experiences and needs, and suggestions for IETP implementation. Quantitative methods will include primary data collection from patients in the IETP at each site to characterize long-term outcomes and patient satisfaction with treatment and secondary data collection to quantitatively characterize patient-level and care system–level data. Finally, data sets will be triangulated to share data findings with partners to inform ongoing implementation efforts.

**Results:**

Data collection began in December 2021 and is currently ongoing. The results and deliverables will inform IETP characterization, evaluation, implementation, and knowledge translation.

**Conclusions:**

The results of this evaluation seek to provide an understanding of the determinants affecting the implementation of IETPs. Service member, staff, and stakeholder insights will inform the state of implementation at each site, and quantitative measures will provide options for standardized outcome measures. This evaluation is expected to inform national Physical Medicine and Rehabilitation Office policies and processes and knowledge translation efforts to improve and expand the IETP. Future work may include cost evaluations and rigorous research, such as randomized controlled trials.

**International Registered Report Identifier (IRRID):**

DERR1-10.2196/44776

## Introduction

### Background

**Figure 1:**
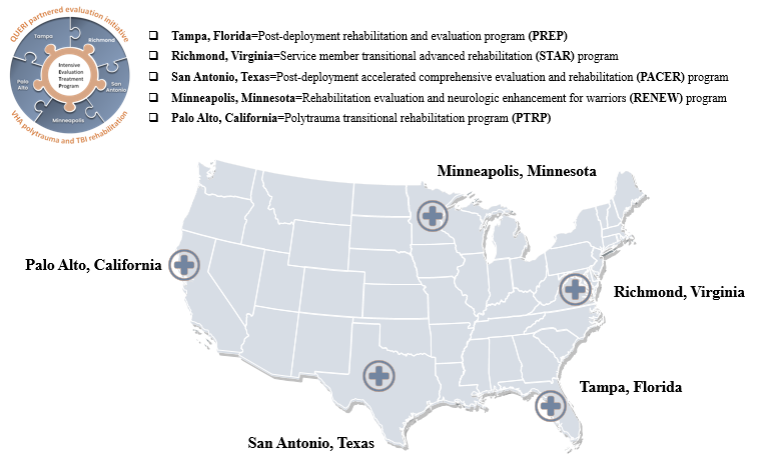
Five Intensive Evaluation and Treatment Program sites. QUERI: Quality Enhancement Research Initiative; TBI: traumatic brain injury; VHA: Veterans Health Administration.

Record numbers of service members with traumatic brain injury (TBI) have flooded the Veterans Affairs (VA) and the Department of Defense (DoD) during the past 2 decades. Since 2000, a total of 430,720 service members have been diagnosed with TBI, and the majority (82.4%) have been diagnosed with mild TBI [1]. Service members consist of conventional forces and special operational forces (SOF). SOF service members undergo extensive training and experience increased risk exposure owing to deployment and job hazards [2]. Sequalae from mild TBI may co-occur with at least 1 of the following: posttraumatic stress disorder (PTSD), chronic pain, and visual and balance disturbances. In particular, the SOF population has a unique presentation of TBI sequala [3]. Interdisciplinary TBI rehabilitation, particularly for the SOF population, is intended to enhance functioning, reduce disability, and improve quality of life. To meet the demand for rehabilitation services required by SOF service members and veterans with TBI, the Veterans Health Administration (VHA) Physical Medicine and Rehabilitation (PM&R) National Program Office developed 5 specialty TBI–Centers of Excellence (TBI-COE) to provide a coordinated approach for comprehensive TBI rehabilitation (Figure 1). The TBI-COE sites are located at VA hospitals in Minneapolis, Minnesota; Palo Alto, California; Richmond, Virginia; San Antonio, Texas; and Tampa, Florida.

To date, there has been no formal characterization or evaluation of the TBI Intensive Evaluation and Treatment Program (IETP) to understand the implementation determinants or formalize core program components across the system of care. This limits the ability of VA to make evidence-based decisions for policy and planning to meet the long-term needs of this patient cohort. To expand the capacity for innovation in delivering guideline-concordant care, the PM&R National Program Office, our operational partner, is supporting a partnered evaluation to implement the effective components of this modality across VHA.

### IETP Partnered Evaluation Goal and Aims

**Figure 2:**
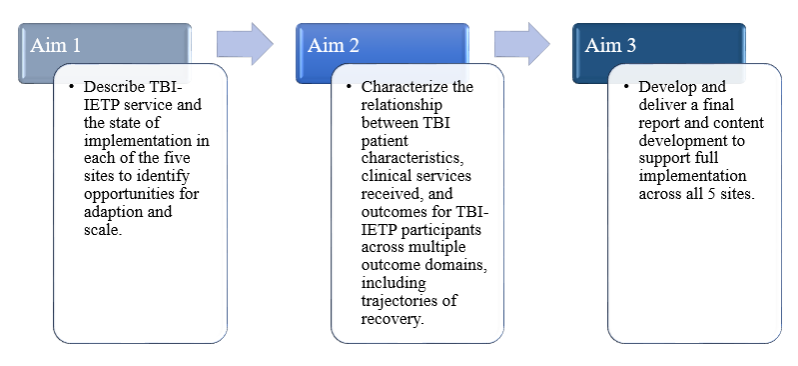
Intensive Evaluation and Treatment Program (IETP) partnered evaluation aims. TBI: traumatic brain injury.

The goal of our partnered evaluation initiative (PEI) is to facilitate the full implementation of the IETP across all 5 VHA TBI-COE sites and to inform minimum standards while supporting the unique characteristics of each site. The aims of this partnered evaluation are as follows: (1) describe IETP services and state of implementation of these services in each of the 5 sites to identify opportunities for adaptation and scale—the findings will identify IETP core aspects and determinants of implementation; (2) characterize the relationship between patient characteristics, clinical services received, and outcomes for participants in the IETP across multiple outcome domains, including trajectories of recovery; and (3) evaluate the clinical impact of IETP components and deliver a final report to include deliverables that support IETP implementation playbook content development (Figure 2). The completion of these aims will promote effective IETP practices across this polytrauma system of care. The development and dissemination of product deliverables from the evaluation will support IETP implementation and continued evaluation over time.

### TBI IETP—Innovative Modalities of Care

Innovative modalities of care improve access to evidence-based care for mild TBI. Innovative models of care have been developed within the polytrauma system of care, which includes 5 specialty TBI-COE that provide comprehensive rehabilitation services for veterans and service members with TBI. The TBI IETP is an innovation that has yielded high consumer demand across locations. The IETP delivers evidence-based care in a residential, inpatient format. The IETP provides bundled evidence-based assessment, treatment, referral, and case management in concordance with the existing guidelines for mild TBI and commonly co-occurring comorbidities (refer to the IETP characterization table in Multimedia Appendix 1 [4-23]). Indeed, the residential format has grown out of necessity to address the unique needs of the SOF population in a compressed, timely manner ideally suited for abbreviated inpatient treatment stays. A recent study by Dismuke-Greer et al [24] highlighted higher annual outpatient use and cost over protracted time intervals for veterans with mild TBI compared with non-TBI controls, highlighting extended intervals and delayed access to evidence-based care. Proponents of IETP emphasize its improved access to evidence-based care, including TBI-adapted treatment of mental health conditions previously published [25,26].

### Responsiveness and Alignment With Goals: Quality Enhancement Research Initiative Implementation Roadmap

#### Overview

In response to this evaluation, engagement with partners and alignment with network director goals will guide implementation across the Quality Enhancement Research Initiative (QUERI) Implementation Roadmap. In response to the special solicitation for a PEI with VHA PM&R, the Characterization, Evaluation, and Implementation of Innovative TBI IETPs will evaluate the ongoing implementation of the IETP at all 5 TBI-COE sites to identify the effective and ineffective components. The project will align with the QUERI Implementation Roadmap, a functional framework for evaluating and contextualizing the implementation of IETP [27]. QUERI Implementation Roadmap elements provide a framework for planning, implementing, and sustaining programs—from reaching audiences to establishing effectiveness, promoting adoption and implementation, and ultimately sustaining maintenance over time. The partner priorities aligned with the roadmap of the framework are summarized in Table 1. Partner goals and challenges will be addressed across the study aims.

**Table 1:** Integration of the Quality Enhancement Research Initiative Implementation Roadmap conceptual model with partner-identified goals, challenges, and partnered evaluation initiative (PEI) solutions.

Aims and partner goals		Partner-identified challenge	PEI solution
**Pre-PEI**			
	Identify and align needs and goals	Work with a team that understands needs and goals from the TBI^a^ and implementation perspective	Ongoing communication and meetings to understand needs and identify priorities
	Engage stakeholders	Prior push to implement not successful	Site representatives recruited to facilitate a bottom-up pull and buy-in from local staff. Input on study design and findings were sought from a stakeholder panel including veterans, service members, VA^b^ and DoD^c^ administrators, and clinicians
	Develop measures and data	Program evaluation data are not standardized and readily accessible	Site representatives recruited to facilitate engagement of stakeholders for data collection. Meetings with PM&R^d^ partners and various stakeholders to identify and prioritize outcomes
**Data to knowledge (preimplementation stage)**			
	Aim 1: characterize consumer demand	IETP^e^ value from referrals within VA and DoD is unknown	Use of qualitative data collection with patients treated across fully and partially implemented sites
	Aims 1-2: fully characterize the innovation (IETP)	IETP is a black box with poor characterization to duplicate and sustain across systems	Use of qualitative interview technique and quantitative analyses of administrative data sets to identify effective practice core elements and adaptation options
	Aim 2: determine early and late outcomes from IETP	Outcome monitoring has not been the focus of the existing programs	Leverage existing data (eg, IETP data) and collect prospective outcome data to identify measures of success and establish baseline performance at the fully implemented site
**Knowledge to implementation (implementation stage)**			
	Aim 1: characterize the degree of implementation	IETP innovations have occurred asynchronously with variation of implementation	Qualitative data collection with a purposive sample of clinicians at each site to understand the degree of implementation, inform selection, and tailor implementation to each site
	Aim 3: disseminate and promote IETP implementation	Continued funding for TBI rehabilitation services in a competitive fiscal environment across the VA organizational hierarchy	Use participatory approach to (1) promote a bottom-up pull for “IETP evidence-based” practices, (2) disseminate implementation content, and (3) gather feedback for each site. Leverage existing reporting structures to summarize findings for reports to Congress including VA and DoD administrators, and clinicians for uptake and sustainment
**Performance to data (sustainment; post-PEI)**			
	Implement IETP	Funding mechanism and timeline insufficient to achieve all partner goals	Will develop future proposals to evaluate the uptake of IETP, evaluate the ongoing implementation, and inform continuous learning

^a^TBI: traumatic brain injury.

^b^VA: Veterans Affairs.

^c^DoD: Department of Defense.

^d^PM&R: Physical Medicine and Rehabilitation.

^e^IETP: Intensive Evaluation and Treatment Program.

#### Preimplementation Stage: Data to Knowledge

Aim 1 qualitative methods will fully characterize the successful IETP model and the perceived value of IETP components. We will leverage existing data resources and prospective data collection to evaluate program outcomes prioritized by our operational partner and other stakeholders, including service members and veterans with mild TBI (aim 2). Findings from the first 2 study aims will be used to develop the foundation for the implementation of effective components and the deimplementation of ineffective components in aim 3.

#### Implementation Stage: Knowledge to Implementation

A participatory approach with IETP stakeholders will be used to develop implementation content that outlines “IETP evidence-based” practices. This effort will involve clinical and administrative IETP stakeholders across study sites as well as a formal stakeholder panel to inform and review a blueprint for implementation.

#### Sustainment: Performance to Data

Future work will address implementation, deimplementation, and their effects on the system and patient outcomes. Table 1 provides an overview of the timing and conduct of the 3 aims.

## Methods

### Partnerships, Research Team, and Relevant Experience

The multiple principal investigators (MPIs) have developed an interdisciplinary team to conduct this mixed methods partnered evaluation with PM&R leadership, informing the development of a stakeholder agenda that is reflected in the aims and deliverables.

**Figure 3:**
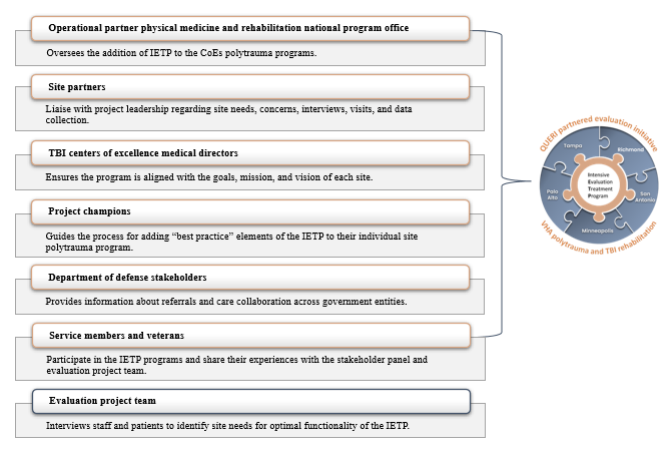
Intensive Evaluation and Treatment Program (IETP) partnered evaluation collaborative roles. COE: Center of Excellence; QUERI: Quality Enhancement Research Initiative; TBI: traumatic brain injury; VHA: Veterans Health Administration.

The PEI engages a stakeholder panel representing multiple stakeholders in IETP from each study site as well as PM&R leadership (Figure 3). The primary goals of the stakeholder panel are to represent operational and clinical perspectives, collaborate on data collection tool development, facilitate site engagement, and identify preferred implementation strategies. Weekly meetings in years 1 through 3 will be held to gather stakeholder input on the study methodology, interpretation of findings, and deliverables.

### Ethical Considerations

The project protocol was reviewed by James A. Haley Veterans’ Hospital Research and Development Committee and deemed *nonresearch*, and no informed consent was necessary. Participants’ identities are confidential, and deidentified data are stored behind VA firewalls. No compensation will be provided to the participants for this program evaluation project. All methods are performed in accordance with the relevant guidelines and regulations. All staff interviews were approved by the VA national union. Patients’ interviews were reviewed by the QUERI administration and were exempt from Code of Federal Regulations Part 1320.3, as the project will evaluate the direct treatment currently provided.

### Aim 1

#### Overview

We will use qualitative methods to describe and characterize IETP to support implementation efforts across all 5 TBI-COE. In alignment with the QUERI Implementation Roadmap, this project will coconstruct a meaningful theoretical context for identifying and describing the implementation of each IETP using the Consolidated Framework for Implementation Research (CFIR) [28,29]. In addition, project leadership will use an implementation evaluation process conducted in a *sister* project, Optimizing Rehabilitation Interventions (ORION) for Cognition Following Complex Traumatic Brain Injury funded by the DoD (W81XWH-19-1-0615), which will maximize the efficiency of this effort and promote interoperability and knowledge translation between VA and DoD. The PEI team will use this implementation evaluation process to present and review the content in initial development in Tampa. Subsequently, the materials will be used and further developed in an iterative fashion at the other IETP sites. The aim 1 deliverable is a characterization of the IETP, IETP Care Implementation Elements Inventory, and IETP Logic Model. The inventory and logic model will be iteratively developed and used to assess the implementation at TBI-COE sites.

#### Sampling and Sample Size

We will collaborate with site partners to recruit a purposive sample of key informants and IETP team members, including a physician, nurse, and therapist, to attend focus groups at each of the 5 sites. As conceptual saturation is the goal, we will recruit the minimum sample necessary to represent the types of professional disciplines to compare experiences. We will also conduct interviews with a purposive sample of DoD IETP stakeholders involved in the referral and coordination of IETP, based on key informant referrals and feedback from our stakeholder panel. As part of a comprehensive evaluation, we will also conduct telephone interviews with service members and veterans from each site who previously participated in IETP, and contact information will be obtained from the data collected for aim 2. Participants will be purposively selected among those in the top and bottom quartiles of the Mayo-Portland Adaptability Inventory (MPAI; patient outcome across all 5 sites) at program discharge [30]. The MPAI reflects the outcomes prioritized by the operational partner. In qualitative projects, sample and size rely on the population characteristics and the quality and richness of information obtained [14,15]. Recruitment efforts will oversample women and minority individuals to ensure that their perspectives are represented in the data. Qualitative recruitment and sampling for aim 1 are presented in Table 2.

**Table 2:** Aim 1 qualitative recruitment.

Data collection activity	Participants	Site sample size:total sample size
Key informant interviews	Clinical team from IETP^a^	1:5
Focus groups	Physician, nurse, therapist, etc	15:75
Interviews	DoD^b^ IETP stakeholders	15:75
Telephone interviews	Service members who previously received IETP	12:60
Demographic questionnaire	Physician, nurse, therapist, etc	15:75
Follow-up interviews	Subsample of key informant and focus group participants	15:75
Site visit observation	IETP representatives, leadership, and clinicians	N/A^c^

^a^IETP: Intensive Evaluation and Treatment Program.

^b^DoD: Department of Defense.

^c^N/A: not applicable.

#### Measures and Data Collection Procedures

We will use site visit observations, key informant interviews, focus group, and follow-up interviews to complete aim 1. We will also refine the fidelity checklist as part of aim 1 data collection (Multimedia Appendix 2). Data collection tools will include veteran and focus group demographic questionnaires (Multimedia Appendix 3), interview and focus group scripts, and a site observation guide and comparison checklist.

#### Initial Interviews

Qualitative researchers will conduct key informant initial interviews remotely with the program or medical director at each site. These participants will be purposively identified and confirmed by the operational partner. Informants will provide an overarching introduction to each site and the site’s implementation efforts and then provide key referrals to recruit other team members for focus group interviews (Multimedia Appendix 4). Key informant interviews will be audio recorded and transcribed verbatim. Researchers will take field notes to capture nuances potentially missed by audio recordings and will facilitate preliminary data analyses when transcription is completed. All data will be stored on a secure VHA server with restricted access. The key informant will also facilitate the analysis of local program outcome data to help recruit local participants in the IETP eligible for telephone interviews.

#### Site Observation

A team of 2 qualitative researchers will travel to each site for site visits. Two-day site visits will comprise (1) a facility tour, (2) presentations from and discussions with program representatives, and (3) meetings with leadership and identified local stakeholders. The qualitative team will take field notes and photos with permission and collect site-specific artifacts. These items will be saved on a secure VHA server with restricted access and uploaded into ATLAS.ti 22 Windows software for organization, coding, and analysis. These partnering activities will be critical for developing relationships with site staff and setting the stage for subsequent aim 3 activities. Site observation guides will be used to assess sites qualitatively, and the checklist will be used in tandem to assess fidelity across sites (Multimedia Appendix 5).

#### Focus Groups

Focus groups, coordinated with the clinical partners to reduce interference with patient care, will be conducted using web conferencing technology to collect data from clinicians about their site’s IETP. Participants will receive an email before the focus group, which will include interview questions. Each focus group will be facilitated by 1 qualitative researcher with a team of evaluation staff to take notes, manage any technological issues, and observe the chat box for any questions or comments. The focus groups will be audio recorded with permission. Information collected during focus groups will address IETP logic, program content, operationalization, and other relevant implementation factors (Multimedia Appendix 6) and will be stored on a secure VHA server with restricted access and uploaded to ATLAS.ti 22 Windows software for organization, coding, and analysis.

#### Follow-up Interviews

Follow-up interviews will be conducted by phone: (1) key informant follow-ups will be conducted to share data summaries, IETP checklists, and draft content and to assess validity and usefulness and (2) focus group member follow-up interviews will be conducted with a subsample of focus group participants to account for differences in knowledge and perspective by role, providing broad perspectives and accounting for potential power differentials. Individual interviews will ensure that team members have an opportunity to address personal perspectives, discuss issues that may not arise in focus groups, explore emergent themes, and review data summaries and content (Multimedia Appendix 7). Follow-up interviews will be conducted via web conferencing and will be audio recorded. Recordings will be transcribed verbatim. Researchers will take field notes to capture nuances potentially missed by audio recordings and facilitate preliminary data analyses when transcription is completed. All data will be stored on a secure VHA server with restricted access.

#### Veteran and Service Member Interviews

Interviews with service members and veterans who participated in IETP will be conducted at each site to describe patient experiences with IETP (Multimedia Appendix 8). These interviews will be audio recorded and transcribed verbatim. Researchers will take field notes to capture nuances potentially missed by audio recordings and facilitate preliminary data analyses when transcription is completed. All data will be stored on a secure VHA server with restricted access.

#### DoD IETP Stakeholder Perspectives

DoD IETP stakeholders will share their perspectives on IETP as consumers to assess the value of VA IETP services from a DoD perspective and obtain their perspectives of important outcomes and their perceptions of the projected trajectory for the continued patient need for IETP services (Multimedia Appendix 9). As with the other qualitative data collection methods, the interviews will be completed using web conferencing and will be audio recorded. Recordings will be transcribed verbatim. Researchers will take field notes to capture nuances potentially missed by audio recordings and facilitate preliminary data analyses when transcription is completed. All data will be stored on a secure VHA server with restricted access.

#### IETP Data Collection Content

In our project start-up, we are developing a preliminary IETP characterization table based on guidelines and subject matter expertise (refer to the preliminary summary in Multimedia Appendix 1). In tandem, using clinical team expertise and literature on evidence-based implementation strategies that are relevant to the IETP clinical components reviewed in the IETP characterization table, the process of implementation to date will be represented as a CFIR-based implementation research logic model (IRLM) [31]. The IRLM will use data on inputs, activities, and outcomes to specify the relationships among determinants of implementation, implementation strategies, and the mechanistic links between strategies and outcomes relevant to the following: (1) inputs—program referral patterns, characteristics of IETP patients, staffing and skills required to deliver the program, facilities, resources needed to deliver the program, and other program inputs evident in the literature review; (2) activities—set of therapeutic activities that comprise the program, characterized as core or peripheral and as individual or group interventions, along with the programmatic meetings and staff training that are necessary to deliver the program; and (3) outcomes—immediate and long-term outcomes that define program success [32]. The IRLM, contextualized by CFIR constructs, will be triangulated and analyzed as part of aim 1 and iteratively revised to reflect the clinical relevance for each site. The team will also refer to the validated inventory of implementation strategies in the development process by Powell et al [33]. The IRLM template is provided in Multimedia Appendix 10 [31].

#### Elicitation of IETP Contents and Operationalization

We will interview respondents to identify the inputs, activities, and outcomes that define the IETP. We will refer to the IETP characterization table to elicit inputs on convergence and divergence between their experience and the guidelines. The preliminary IETP characterization table will be used as a plausibility check for these initial interviews to evaluate, for instance, whether the elicited program inputs, activities, and outcomes are concordant with the published literature and whether the suggested links between program activities and outcomes are plausible. The analysis will be conducted within the context of CFIR to ensure strategic alignment with the implementation recommendations. The elicitation of program inputs, activities, and outcomes will be expedited by comparing similar products developed in the ORION project. ORION project library elements that correspond to IETP inputs, activities, and outcomes will be shared with the interviewees as examples. This process will support the iterative development of the IETP Care Implementation Elements Inventory. Multimedia Appendix 11 provides an example from the ORION project.

#### Data Analysis

Qualitative data collection and analysis will occur concurrently. Insights from the data analysis will be used to iteratively guide data collection. The evaluation team will work together to develop an a priori code book based on the 2009 CFIR constructs, as each interview guide is also based on the 2009 CFIR constructs. Transcripts, artifacts, and observation notes will be uploaded to ATLAS.ti 22 Windows software to organize and code data. Analysis methods will be used to identify domains related to data for specific data types, such as key informant interviews and focus groups [34]. Participant comments will be organized to develop codes and merged to develop categories. Categories will be compared, and relationships will be identified. Data samples will be extracted and coded by 2 project team members and evaluated for interrater reliability and credibility. The data sets for each site will be compared to determine the commonalities and differences between sites. The evaluation team will conduct a matrix analysis to analyze across-site domains and taxonomies [35]. Descriptive and comparative matrices will identify the patterns of regularities and inconsistencies and identify the relevant and representative components by site to support strategy development. Once cases and comparative matrices are developed, follow-up interviews will be reviewed by the stakeholder panel to verify the findings and provide additional input and clarification.

### Aim 2

Aim 2 will use the existing data to identify IETP participants; types of care received; and long-term outcomes, including postdischarge needs and trajectories, of IETP. The models will be reviewed with clinical partners throughout, with the final analytic output delivered at the end of year 2.

#### Measures and Data Collection Procedures

Data from each IETP participant will be obtained from site leads beginning with Tampa (2015 to present; n=approximately 350). We will obtain identifiers that allow linkage to VA and DoD health system data and create a study crosswalk database behind the VA firewall that includes the encrypted identifier, a study ID, and contact information for interview and survey administration. We will obtain intake and discharge measures from each IETP site. We will assess IETP fidelity based on the IETP characterization table (Multimedia Appendix 1) using a checklist (Multimedia Appendix 2) and chart abstraction focusing on the admission note, admission IETP care plan, discharge TBI care plan, and discharge summary, which are highly accurate in describing the reasons for seeking treatment, baseline status, the type of treatment recommended, the type of treatment received, and outcomes on discharge (Multimedia Appendix 12).

We will also link these IETP data to VA and DoD health system data, as we have done previously [36], to obtain information on physical and mental health, medications, and military demographic data before and after IETP. As approximately 90% (225/250) of participants in the IETP are active duty at the time of treatment with the goal of returning to full duty and operational partner priority outcomes include return to duty or employment, the use of VHA data (eg, Patient Health Questionnaire-9 [PHQ-9] and PTSD checklist-5) is not a feasible approach to long-term outcomes. Therefore, we will conduct a survey using a VA-approved web-based survey software for participants of IETP (2015-2022; approximately 500) using a Modified Dillman Method, which we used in prior VA studies (Multimedia Appendix 13) [37-40]. We will begin a survey of earlier IETP graduates in the first year of the study and send surveys to new graduates 6-9 months post study completion. The final outcome measures included in the survey will be developed in collaboration with stakeholders. The current list of stakeholder-identified measures that are evaluated at intake and discharge include the PHQ-9, PTSD checklist-5, MPAI, and the Neurobehavioral Symptom Inventory.

We will also use VA and DoD health system data to describe health system outcomes such as use, new diagnoses, and adverse outcomes, which will, together with data baseline, discharge, fidelity, and discharge outcomes, be used to develop trajectories of recovery.

#### Characterize IETP Participants

We will begin characterization with the Tampa site (n=approximately 350) and Richmond site (n=approximately 150), followed by other sites. We will use VA and DoD health system data to characterize preadmission military and sociodemographic characteristics, TBI-related diagnoses (eg, PTSD, pain, and sleep), and medication use (eg, opioids, antidepressants, and sedative hypnotics). We will add data from TBI history and symptom inventories administered at IETP admission to develop mild TBI phenotypes [36,41,42]. This allows the characterization of participants in the IETP and the identification of groups that are likely to have similar courses of care.

#### Data Analysis

After calculating descriptive statistics (proportions and quantiles for discrete variables and means and SDs for continuous variables) of VHA and DoD intake measures before IETP admission, we will fit a latent variable mixture model, a multivariate statistical technique used to classify individuals into interpretable categories based on a set of measurements (described in the *Measures and Data Collection Procedures* section) [36,42-49]. Similar to cluster analyses, the latent variable mixture model is an exploratory technique to identify underlying constructs associated with multiple variables, in our case, the underlying comorbidity phenotypes. On the basis of prior work, we expect 2 to 3 groups each with n=approximately 100-200 participants [36]. Health system data identifying the treatment provided are not likely to have missing data owing to the professional responsibility to document. Each visit requires a primary diagnosis for care, which will lead to data available at each visit; therefore, we expect little to no missing data.

#### Identify Treatment Received During IETP

Consistent with prior studies [50-52], we will create an abstraction tool in collaboration with IETP clinicians and aim 1 data. Using the crosswalk database, we will conduct chart abstraction for IETP focusing on the initial assessment, TBI care plan, processes and amount of care received, discharge assessment, and TBI care plan. After finalizing the procedures and appropriate training, team members will independently review 10 charts and then meet to identify inconsistencies, errors, or disagreements and resolve any by consensus. This will continue until there is 90% agreement on all items abstracted, to ensure abstraction fidelity. The areas of uncertainty will be discussed with IETP clinicians. These data will be merged with IETP data, VA and DoD analytic file data, and survey data for the outcome analyses.

We will identify what happens during the IETP stay using descriptive statistics for the overall cohort, stratified by phenotypes from the measures listed in the *Measures and Data Collection Procedures* section. We expect that different types of care will be provided to individuals with different prior comorbidities and clinical presentations. We will apply Pearson chi-square test to assess the association between recommendations for admission TBI care plan and TBI phenotype.

#### Identify Components of IETP Associated With Sustainment and Improvement Trajectories

Outcomes currently identified by operational and clinical stakeholders that are collected at all sites (Tables 3 and 4) will be included in the survey to develop longitudinal outcome trajectories. On the basis of stakeholder feedback, we will also measure and describe community reintegration, employment, retention in the military where relevant, compliance to discharge from the TBI care plan, reasons for difficulty in care plan compliance, and unmet rehabilitation needs. Per operational partners’ request, we will also use VHA and DoD data to identify use relevant to discharge (eg, TBI care plan within primary, mental health, specialty care, or emergency care).

We will use Bayesian network analysis to generate a directed acyclic graph, which probabilistically describes the trajectory of symptoms, care, and outcomes for IETP participants. This network will enable us to explore the contribution of the baseline phenotype to the decision to use a particular treatment course and the contribution of those treatments to the participants’ resulting outcome measures [53]. Using a hill-climbing algorithm scored with the Bayesian information criterion, we will establish directional connections between nodes, called edges, and evaluate the robustness of the edges using bootstrapped samples of the data. We will a priori exclude any implausible directed edges (eg, IETP care cannot lead to precare symptoms). The resulting directed network will have edges between the levels: (1) phenotypes, (2) treatment, and (3) outcomes, while disallowing connections that go from levels 2 to 1 or 3 to 2 or 1. We can allow edges between nodes within the treatment level of the network to better understand the interactions between treatments and to assess how the treatments individually and in combination probabilistically affect patient outcomes. By fitting this model, we can isolate the most probable trajectories between TBI phenotypes and treatments and then determine which treatments have a higher probability of positive long-term outcomes. To gain an understanding of the treatment courses on outcomes, we will present a graphical display of the nodes and edges, the conditional probabilities calculated between nodes that estimate the probability of long-term outcomes given the participants’ IETP components and comorbidity phenotypes, and the robustness measures from bootstrapping.

**Table 3:** Proposed measures for developing trajectory outcomes.

Measure	Baseline	Discharge	Survey	Construct
Mayo-Portland Adaptability Inventory	✓	✓	✓	Ability, adjustment, and participation
Patient Health Questionnaire-9	✓	✓	✓	Depression
PTSD^a^ checklist-5	✓	✓	✓	PTSD
Neurobehavioral Symptom Inventory	✓	✓	✓	Postconcussion symptoms

^a^PTSD: posttraumatic stress disorder.

**Table 4:** Proposed measures for developing trajectory outcomes.

Health system data	Prebaseline	During IETP^a^	After IETP	Construct
Diagnoses	✓	✓	✓	History and emergence of physical or psychiatric comorbidity
Health care use	✓	✓	✓	Type of care received
Medications and procedures	✓	✓	✓	Type of pharmacological or nonpharmacological care

^a^IETP: Intensive Evaluation and Treatment Program.

#### Power and Missing Data

The analyses in this aim take an exploratory approach to establishing phenotypes with latent variable mixture models and isolating common trajectories based on Bayesian networks. As such, we do not have a targeted hypothesis with which to power this analysis for minimum detectable effect sizes but rather a model selection approach using the Bayesian information criterion. With 500 participants, we expect that our sample size will be well above the number of nodes in the Bayesian network, which will allow for an accurate estimation of the conditional probabilities in the network [54,55].

We will assess the risk of bias owing to missing data by looking at patterns of missing measurements at each phase of the patients’ trajectories, although we expect most missingness to be postdischarge. Reasons for missing measurements will be tabulated with a focus on differentiating missed measurements for logistical reasons from dropouts or intermittent missingness potentially related to the condition of the patient. If >10% (25/250) of the patients have missing outcome measurements for any of the follow-up measures or if the comparisons of baseline characteristics between patients with missing and nonmissing measurements indicate considerable imbalances, rather than only looking at complete cases, we will apply the structural expectation-maximization algorithm, an imputation method that directly processes data with missing values while fitting the Bayesian network [56]. This method incorporates baseline and follow-up factors beyond the variable being analyzed to account for the dependence of missing data on other factors. TBI history is a primary piece of data required for admission to the program. It is unlikely that any data will be missing for TBI history. However, if >20% (>50/250) of the sample has missing data related to TBI history, the study should be stopped.

### Aim 3

#### Overview

In aim 3, aim 1 and aim 2 data will be triangulated to develop a final report to support IETP full implementation at sites. The primary deliverable for aim 3 is a final report that will include the IETP characterization table, IETP Care Implementation Elements Inventory, IRLM, patient experience data summaries, implementation checklists, educational content, and templates. A concluding presentation will be delivered, and consultation on findings integration will be provided for each site. In year 3, we will conduct site visits and presentations with each program to present the developed content and consult with each site to individualize and prioritize how content can be tailored at each site to facilitate full IETP implementation, including the deimplementation of ineffective care.

#### Measures and Data Collection Procedures

For the purposes of this PEI, the final report will be delivered to operational partners as both (1) a tool that can be used at sites to strategize the implementation of effective IETP practices and deimplementation of low-value care and (2) a resource that will include education about research implementation to support knowledge translation.

The team will develop a product grid to propose and prioritize implementation content with the stakeholder panel. Prioritized products and content from the product grid will be developed and packaged into a comprehensive report. Although the team will leverage previous projects to expedite the PEI efforts, the products will be unique to this evaluation. The final report content will be developed iteratively, and components will be reviewed with participants throughout the PEI to ensure quality control and fidelity of the developed content. In addition, the team and stakeholder panel will attend a meeting in year 3 to review and validate the IETP content. The engagement of other key stakeholder groups will be determined based on data collected in aims 1 and 2. Potential groups include consultation specialists in sleep and pain, rehabilitation disciplines, and program administrators.

Characterizing the core components of IETP as a fully implemented program will inform subsequent staged implementation efforts across sites by establishing means for monitoring the fidelity, timing, and teaching of programs over time. Products will not be designed to clone IETP across sites but will rather support site-to-site harmonization and implementation, sustainment, or enhancement of key components. Although we have established a strategic plan for acquiring data to inform product development, to be stakeholder driven, we contend that to fully identify aim 3 activities before aim 1 and aim 2 data collection would be premature. Identifying implementation efforts, including best practices, facilitators, barriers, strengths, and challenges, will be a solid approach to developing tailored implementation content.

## Results

Funding and start-up activities for the evaluation began in April 2021, and the evaluation officially began in October 2021. Data collection is underway at all 5 sites and is expected to continue through May of 2025. Key informant and staff focus groups have been completed at 4 of 5 sites; the fifth site was launched in April of 2023. DoD and patient interviews are underway at all 5 sites, with one site observation to be conducted. Data analyses are underway. The anticipated evaluation results will be disseminated by 2024 and thereafter.

## Discussion

### Principal Findings

The goal of this evaluation protocol is to facilitate the full implementation of IETP at all VA TBI-COE sites and to inform minimum standards while supporting the unique characteristics of each site. In alignment with the goals of the proposal, ineffective treatment components will be targeted for deimplementation. The findings of this mixed methods evaluation effort will allow the characterization, evaluation, and implementation of IETP across the VA polytrauma system to improve the standardization and fidelity of care for SOF service members and veterans with mild TBI and co-occurring conditions, while preserving the unique facets of the individual program sites.

### Potential Limitations

The proposed PEI does not address implementation sustainment and cost. Implementation and cost evaluation elements are beyond the scope of this 3-year PEI; however, the proposed aims are foundational efforts that will facilitate a cost and sustainment evaluation after the proposed PEI. A “rigorous” design (eg, step wedge, randomized controlled trial) is not proposed. Our proposed mixed methods design is aligned with the preimplementation emphasis of operational requests and needs. There is a potential lack of clinician or administrator participation in aim 1. We partnered with coinvestigators at each study site to support recruitment. We will meet with them during “training time” or “clinical team meetings” to maximize their availability and convenience to participate. We will offer individual interviews (by phone or in person) to maximize participation for those unable to participate in the focus groups. We will conduct meetings in person or by phone to meet their preferences, particularly through individual interviews. All patients will be interviewed by phone. The time frame is compressed for study activities. For aim 1, we will conduct simultaneous site visits and rapid iterative analysis as data are collected and analyzed. Transcription turnaround time is minimized using an internal transcription service with an established protocol with the Tampa team. For aim 2, we will leverage existing data sets (i.e, IETP, TBI model systems, VA, and DoD data sets) and include site leads as experts in those data sets to facilitate timely analyses. A potential pitfall of aim 2 is the lack of participation in long-term outcome surveys; we will address this with an introductory letter from PM&R describing the importance of the study, as well as individualized follow-up strategies that have been effectively used by the team in previous projects. For aim 3, final report content development will begin at the onset of the PEI, supported throughout aims 1 and 2, relying on protocols and templates used in other projects. We will use an iterative design process that will support the development and validation of the content with the PEI stakeholder panel. There is no comparison group for this treatment modality. The operational partner did not want to prioritize this comparison and felt this would be better suited in subsequent proposals.

### Management Plan and Timeline

This team initiated a comprehensive approach with operational partners to ensure timely deliverables. Operational partners have described their expectations and anticipated deliverables; as such, they informed and approved the proposed evaluation and activities. The proposed aims represent distinct deliverables as a result from each aim, which are to be delivered within the established project timeline (Multimedia Appendix 14). In addition to deliverables, the PEI team will provide operations with quarterly reports to include current tasks; progress; recruitment, data collection, and analysis benchmarks; PEI barriers and solutions; product development; and lessons learned.

### Communication and Collaboration

This team has established effective communication and collaboration across sites to allow visual and audio access and secure shared folder and drive access. We will use 4 primary means of project management and communication to complete the study within the projected timeline. First, we conduct weekly meetings, attended via web-based platform by the team from the primary site, including MPIs, coinvestigators, and project managers, to address day-to-day activities. The MPIs also conduct weekly meetings with their operational partners. Second, at the onset of the study, the PEI team, site leads, and operational partners will attend a virtual kick-off meeting. At this kick-off, we will attend to start-up issues and collaborate with operational partners to confirm and refine needs and anticipated deliverables. As a result of this kick-off meeting, the team will initiate a Memorandum of Understanding outlining specific deliverables. The Memorandum of Understanding is a standard agreement between operational partners and research teams to establish expectations and performance outcomes relevant to project aims. Third, the team, including the stakeholder panel, will attend quarterly meetings with relevant agenda topics to address evaluation activities, benchmarks, project issues, and subsequent implementation efforts. We propose quarterly meetings with PEI team members and operations partners to facilitate ongoing data reports, operational updates, immediate reporting and resolution of any PEI barriers and concerns, and presentation of deliverables. This ongoing communication will facilitate any changes or operations-prompted amendments to the PEI, as warranted by operational partners. In this case, MPIs will consult with operations and reconceptualize project activities and deliverables as needed. As needed, updates will be made on the weekly meeting calls and email correspondence. Updates will be summarized in quarterly reports to the operational partners.

### Conclusions

This PEI will examine the implementation of the IETP across 5 sites and characterize program participants, core components, and unique site adaptions. Components that are associated with improved participant outcome trajectories will be identified. This information will be the foundation for the future implementation of effective IETP components and deimplementation of ineffective components. Future work may include cost evaluations and rigorous research such as randomized controlled trials.

## Appendix

Multimedia Appendix 1 Intensive Evaluation and Treatment Program characterization table.

Multimedia Appendix 2 Intensive Evaluation and Treatment Program fidelity checklist.

Multimedia Appendix 3 Intensive Evaluation and Treatment Program demographic questionnaire.

Multimedia Appendix 4 Intensive Evaluation and Treatment Program key informant interview script.

Multimedia Appendix 5 Intensive Evaluation and Treatment Program site observations guide.

Multimedia Appendix 6 Intensive Evaluation and Treatment Program focus group guide.

Multimedia Appendix 7 Intensive Evaluation and Treatment Program follow-up interviews script.

Multimedia Appendix 8 Intensive Evaluation and Treatment Program veteran and service member interview script.

Multimedia Appendix 9 Intensive Evaluation and Treatment Program Department of Defense stakeholder interview script.

Multimedia Appendix 10 Intensive Evaluation and Treatment Program implementation research logic model.

Multimedia Appendix 11 Intensive Evaluation and Treatment Program implementation elements.

Multimedia Appendix 12 Intensive Evaluation and Treatment Program chart abstraction.

Multimedia Appendix 13 Intensive Evaluation and Treatment Program long-term outcomes.

Multimedia Appendix 14 Gantt chart of project activities across the 3-year project.

## Abbreviations

CFIR: Consolidated Framework for Implementation Research
DoD: Department of Defense
IETP: Intensive Evaluation and Treatment Program
IRLM: implementation research logic model
MPAI: Mayo-Portland Adaptability Inventory
MPI: multiple principal investigator
ORION: Optimizing Rehabilitation Interventions
PEI: partnered evaluation initiative
PHQ-9: Patient Health Questionnaire-9
PM&R: Physical Medicine and Rehabilitation
PTSD: posttraumatic stress disorder
QUERI: Quality Enhancement Research Initiative
SOF: special operational forces
TBI: traumatic brain injury
TBI-COE: Traumatic Brain Injury–Centers of Excellence
VA: Veterans Affairs
VHA: Veterans Health Administration

## Data Availability

The data for this project are available from the corresponding author upon reasonable request.
