# Supplementary material for: Traumatic Brain Injury Intensive Evaluation and Treatment Program: Protocol for a Partnered Evaluation Initiative Mixed Methods Study
Source: JMIR Res Protoc. 2023 May 9;12:e44776. doi: 10.2196/44776 (PMC10206625; doi:10.2196/44776)
Supplement: Multimedia Appendix 1 [file resprot_v12i1e44776_app1.pdf]

Appendix 1. IETP Characterization Table: Summary of IETP program evidence-based and clinician self-reported practice-based interventions					
**Domains Addressed in the VA/DOD Mild TBI Guidelines**	**Actor**	**Actions During Tampa IETP Inpatient Program**			**Evidence-Base** *(see legend)*
	Provider	Assessment	Treatment	Referral	4, 5, 8
**Headache**	A	✓	✓	✓	5, 6
	B	✓	✓		
	B			✓	
	H	✓	✓	✓	12
	F	✓	✓	✓	
	E	✓	✓	✓	4, 6, 15, 18
**Other Pain**	A	✓		✓	
	B	✓	✓		11
	B			✓	
	H	✓	✓	✓	12
	F				
	E	✓	✓	✓	18
**Dizziness and Disequilibrium**	B			✓	
	G		✓		
	E	✓	✓	✓	16, 17
	F	✓	✓	✓	
	E	✓	✓	✓	14, 16, 17, 23
**Tinnitus**	B			✓	
	E	✓			
	H	✓	✓	✓	12, 13
	E	✓	✓	✓	4, 18, 23
**Sleep**	A	✓	✓	✓	5

	B	✓			5
	B			✓	
	E	✓	✓	✓	5
	F	✓	✓	✓	
	E			✓	4, 18
Insomnia	B	✓	✓		
Sleep Apnea	A	✓	✓	✓	11
Other Sleep Disorder	H	✓	✓	✓	20
**Cognition**	A	✓	✓	✓	4
	B			✓	4
	H	✓	✓	✓	12
	F	✓	✓	✓	
	D	✓	✓	✓	4, 19, 23
	E	✓	✓	✓	4, 18
	C	✓	✓		4
**Depression**	A	✓	✓		
	B	✓	✓		8
	H	✓	✓	✓	12
	F	✓	✓	✓	
	E	✓		✓	18
**PTSD**	B	✓	✓		7
	B	✓	✓		7
	H	✓	✓	✓	12, 23
	F	✓	✓	✓	
**Substance Use Disorders**	A	✓	✓		
	B	✓		✓	9
	B		✓	✓	9
	H		✓	✓	12
	E	✓	✓	✓	9

	F	✓	✓	✓	
	E			✓	
**Suicidality**	A	✓	✓		4
	B	✓			4
	B	✓	✓	✓	10
	H	✓	✓	✓	10
	E	✓		✓	
	C	✓			
**Other:** (specify)____________	B	✓			
	B			✓	7
	H	✓	✓	✓	22
Anger	E	✓	✓	✓	
Bladder	E			✓	
Marriage	E			✓	
Leisure	F	✓	✓	✓	

Legend Denoting Program Providers: A=physician, B=psychologist, C=neuropsychologist, D=SLP, E=PT, E=OT, F=Rec Therapy, G=Nursing, H=Art Therapy, I=Other.

Legend Denoting Evidence:

4. VA/DoD Clinical Practice Guideline for the Management of Concussion-Mild Traumatic Brain Injury. VA DoD Clinical Practice Guidelines Website. https://www.healthquality.va.gov/guidelines/rehab/mtbi/index.asp. Accessed May 24, 2021.
5. VA/DoD Clinical Practice Guideline for the Management of Chronic Insomnia and Obstructive Sleep Apnea. VA DoD Clinical Practice Guidelines Website. https://www.healthquality.va.gov/guidelines/CD/insomnia/index.asp. Accessed May 24, 2021.
6. VA/DoD Clinical Practice Guideline for the Primary Care Management of Headache. VA DoD Clinical Practice Guidelines Website. https://www.healthquality.va.gov/guidelines/Pain/headache/. Accessed May 24, 2021.
7. VA/DoD Clinical Practice Guideline for the Management of Posttraumatic Stress Disorder and Acute Stress Disorder. DoD Clinical Practice Guidelines Website. https://www.healthquality.va.gov/guidelines/MH/ptsd/. Accessed May 24, 2021.
8. VA/DoD Clinical Practice Guideline for the Management of Major Depressive Disorder. DoD Clinical Practice Guidelines Website. https://www.healthquality.va.gov/guidelines/MH/mdd/. Accessed May 24, 2021.
9. VA/DoD Clinical Practice Guideline for the Management of Substance Use Disorders. DoD Clinical Practice Guidelines Website. https://www.healthquality.va.gov/guidelines/MH/sud/. Accessed May 24, 2021.
10. VA/DoD Clinical Practice Guideline for the Assessment and Management of Patients At-Risk for Suicide. DoD Clinical Practice Guidelines Website. https://www.healthquality.va.gov/guidelines/mh/srb/index.asp. Accessed May 24, 2021.

11. VA/DoD Clinical Practice Guidelines for Opioid Therapy for Chronic Pain. https://www.healthquality.va.gov/guidelines/Pain/cot/VADoDOTCPG022717.pdf. Accessed May 25, 2021.
12. National Endowment for the Arts. Creative Forces Clinical Research: A Strategic Framework and Five-Year Agenda (2018-2022). https://www.arts.gov/sites/default/files/CF-Clinical-Research-Framework-and-Agenda-10.23.18.pdf. Accessed May 22, 2021.
13. Michigan Occupational Therapy Association (MiOTA). Assessment and intervention of visual perception and cognition following brain injury and the impact of everyday functioning. https://miota.org/docs/Assessment_and_Intervention_of_Visual_Perception_and_Cognition3.pdf. Accessed May 21, 2021.
14. American Speech Language Hearing Association. Clinician's Guide to Cognitive Rehabilitation in Mild Traumatic Brain Injury: Application for Military Service Members and Veterans. https://www.asha.org/siteassets/practice-portal/traumatic-brain-injury-adult/clinicians-guide-to-cognitive-rehabilitation-in-mild-traumatic-brain-injury.pdf. Accessed May 22, 2021.
15. Headache Classification Committee of the International Headache Society. The International Classification of Headache Disorders 3rd Edition. https://ichd-3.org/wp-content/uploads/2018/01/The-International-Classification-of-Headache-Disorders-3rd-Edition-2018.pdf. Accessed May 25, 2021.
16. American Physical Therapy Association – Neurology Section. Vestibular Rehabilitation for Peripheral Vestibular Hypofunction: An Evidence-Based Clinical Practice Guideline. Website. https://www.neuropt.org/practice-resources/anpt-clinical-practice-guidelines/vestibular-hypofunction-cpg. Accessed May 25, 2021.
17. Bhattacharyya N, et al. Clinical practice guideline: Benign paroxysmal positional vertigo (Update). *Otolaryngol Head Neck Surg*. 2008;139(5 Suppl 4):S47-81. PMID: 18973840.
18. Quatman-Yates CC, et al. Physical therapy evaluation and treatment after concussion/mild traumatic brain injury: Clinical practice guidelines linked to the International Classification of Functioning, Disability and Health From the Academy of Orthopaedic Physical Therapy, American Academy of Sports Physical Therapy, Academy of Neurologic Physical Therapy, and Academy of Pediatric Physical Therapy of the American Physical Therapy Association. *J Ortho Sports Phys Ther*. 2020;50(4):CPG1-CPG73. DOI: 10.2519/jospt.2020.0301
19. Silverberg ND, Iverson GL, on Behalf of the on behalf of the ACRM Mild TBI Definition Expert Consensus Group and the ACRM Brain Injury Special Interest Group Mild TBI Task Force. Expert panel survey to update the American Congress of Rehabilitation Medicine definition of mild traumatic brain injury. *Arch Phys Med Rehabil*. 2021;102(1):76-86. DOI: 10.1016/j.apmr.2020.08.022

<u>Secondary Evidence</u>:

20. Aurora RN, Zak RS, Auerbach SH, et al. Best practice guide for the treatment of nightmare disorder in adults. *J Clin Sleep Med.* 2010;6(4):389-401. DOI: 10.5664/jcsm.27883.
21. Kaimal G, Jones JP, Dieterich-Hartwell R, Acharya B, Wang X. Evaluation of long- and short-term art therapy interventions in an integrative care setting for military service members with post-traumatic stress and traumatic brain injury. *Art Psychother*. 2019;62:28-36. DOI: 10.1016/j.aip.2018.10.003.
22. Garnett C, Spiro N, West R, Müllensiefen D. How do artistic creative activities regulate our emotions? Validation of the Emotion Regulation Strategies for Artistic Creative Activities Scale (ERS-ACA). *PloS One*. 2019;14(2):e0211362. DOI: 10.1371/journal.pone.0211362.
23. Ellis MJ, Leddy JJ, Willer B. Physiological, vestibulo-ocular and cervicogenic post-concussion disorders: An evidence-based classification system with directions for treatment. *Brain Inj*. 2015;29(2):238-48. DOI: 10.3109/02699052.2014.965207.
