# Supplementary material for: Traumatic Brain Injury Intensive Evaluation and Treatment Program: Protocol for a Partnered Evaluation Initiative Mixed Methods Study
Source: JMIR Res Protoc. 2023 May 9;12:e44776. doi: 10.2196/44776 (PMC10206625; doi:10.2196/44776)
Supplement: Multimedia Appendix 2 [file resprot_v12i1e44776_app2.pdf]

Appendix 2. Mild TBI Guideline Fidelity Checklist Adapted for TBI-IETP Programs Treating Veterans and Service Members with History of Mild TBI and Persistent Symptoms				
Domain/Recommendation	Procedure	Schedule	Discipline	Comment
**A. Diagnosis and Assessment**				
1. Language recommendation in communication with patients "history of mild TBI"	NA	__On admission<br>__Daily<br>__Weekly<br>__At discharge<br>Other	__MD<br>__Neuro/Psych<br>__SLP<br>__OT<br>__PT<br>__Rec<br>Other	Not reliably available for chart abstraction
2. Evaluating individuals presenting with symptoms at initial presentation	NA		__MD<br>__Neuro/Psych<br>__SLP<br>__OT<br>__PT<br>__Rec<br>Other	Intake for IETP including IETP Primary measures: MPAI, PHQ-9, PCL-5, NSI<br>Others based on complaints are documented
3. For those in the post-deployment setting, do not use neuroimaging, serum biomarkers, or EEG to establish a diagnosis of mTBI or to establish care.	NA	NA	NA	-identify neuroimaging, serum biomarkers/ EEG and reason for use. Note if using to establish diagnosis of mTBI versus symptomatic reason (e.g. epilepsy)
4. No neuropsychological testing within first 30 days following mTBI.				Document use of neuropsychological tests and time since TBI; identify those <30 days
5. In the post-deployment setting, do not use ANAM, NCAT, or ImPACT in routine care.	NA	NA	NA	If done, by whom (to target de-implementation efforts)?
6. For those with developing new symptoms greater than 30 days post mTBI, administer a focused diagnostic workup specific to symptom complaints.	Administer focused diagnostic workup for specific complaints.		__MD<br>__Neuro/Psych<br>__SLP<br>__OT<br>__PT<br>__Rec<br>__Other	Intake for IETP including IETP Primary measures: MPAI, PHQ-9, PCL-5, NSI<br>Others based on complaints

**B. Co-occurring Conditions**				
7. Individuals with persistent mTBI symptoms should receive psychiatric evaluation for major depressive disorder, post-traumatic stress disorder, substance use disorder, and suicidality. *[follow guidance in VA/DoD clinical guidelines for these conditions]*	Administer psychiatric evaluation for specific conditions/symptoms		__MD<br>__Neuro/Psych<br>__SLP<br>__OT<br>__PT<br>__Rec<br>__Other	-Intake for IETP including Primary measures regarding mental health symptoms: PHQ-9; GAD-2 or GAD-7, PCL.<br>-Evaluate: Refer to behavioral health provider.<br>-Assess for SI/HI.<br>-Evaluation should include lack of psychosocial support, negative illness expectations, and Compensation/litigation.
**C. Treatment**				
8. Offering a primary care, symptom driven approach in the E&M of patients with history of mTBI and persistent symptoms.	Provide comprehensive E&M addressing symptoms and comorbidities below.	NA	__MD<br>__Neuro/Psych<br>__SLP<br>__OT<br>__PT<br>__Rec<br>Other	Evaluate primary IETP measures over time to assess for change in symptoms over treatment MPAI, PHQ-9, PCL-5, NSI
**a. Effect of mTBI Etiology on Treatment Options and Outcomes**				
9. Treatment should not be adjusted based on mechanism of injury.	NA	NA	NA	NA
10. Prognosis is not adjusted based on mechanism of injury.	NA	NA	NA	NA
**b. Headache**				
11a. Headache education is provided and includes stimulus control, use of caffeine/tobacco/alcohol and other stimulants.	Provide headache education as needed per symptoms.	NA	__MD<br>__Neuro/Psych<br>__SLP<br>__OT<br>__PT<br>__Rec<br>Other	-Headache education documented
11b. Non-pharmacologic interventions are offered for headache including sleep hygiene, dietary modification, physical therapy, relaxation, and modification of the environment.	Obtain a sleep history, evaluate diet, prescribe PT, prescribe relaxation, and identify and modify environmental triggers.	NA	__MD<br>__Neuro/Psych<br>__SLP<br>__OT<br>__PT<br>__Rec<br>__Other	-Identify non-pharmacologic interventions offered and provided (sleep history, evaluate diet, prescribe PT, prescribe relaxation, and identify and modify environmental triggers)
11c. Pharmacologic interventions for acute HA and prevention of attacks.	See Table B-3 and B4 for medication guidance; refer to specialist if ineffective.	NA	__MD<br>__Neuro/Psych<br>__SLP<br>__OT<br>__PT<br>__Rec<br>Other	Identify pharmacological treatment provided for headache and identify concordance/discordance with guideline recommendations.

**c. Dizziness and Disequilibrium**				
12. Refer or offer short-term trial of vestibular, visual, and proprioceptive exercise offered for those with symptoms and functional impairment.	See Table B-5 for referral criteria.	NA	__MD<br>__Neuro/Psych<br>__SLP<br>__OT<br>__PT<br>__Rec<br>__Other	Identify pharmacologic treatments:<br>Pharmacologic intervention not indicated outside of the acute period.<br>Referral to qualified vestibular therapist for persistent symptoms.
**d. Tinnitus**				
13. No intervention recommended	NA	NA	__MD<br>__Neuro/Psych<br>__SLP<br>__OT<br>__PT<br>__Rec<br>Other	-identify treatment provided for tinnitus
**e. Visual Symptoms**				
14. No intervention recommended for visual symptoms, diplopia, accommodation or convergence disorder, or visual tracking deficits.	NA	NA	__MD<br>__Neuro/Psych<br>__SLP<br>__OT<br>__PT<br>__Rec<br>__Other	- identify treatment provided for visual symptoms, diplopia, accommodation or convergence disorder, or visual tracking deficits.
**f. Sleep**				
15a. Sleep education should be provided as indicated including sleep hygiene, stimulus control, use of caffeine/tobacco/ alcohol/ and other stimulants.	Sleep education provided	NA	__MD<br>__Neuro/Psych<br>__SLP<br>__OT<br>__PT<br>__Rec<br>__Other	Identify specific types of sleep education provided (e.g. sleep hygiene, stimulus control, use of caffeine/tobacco/alcohol/ and other stimulants).
15b. Non-pharmacologic interventions offered as indicated.	Non-pharmacologcal interventions provided as needed.	NA	__MD<br>__Neuro/Psych<br>__SLP<br>__OT<br>__PT<br>__Rec<br>Other	Identify non-pharmacological treatments provided for sleep (e.g. CBT for insomnia, CPAP, etc.)
15c. Pharmacologic interventions offered to aid sleep initiation and maintenance as indicated.	Pharmacological interventions consistent with sleep guidelines provided as needed.	NA	__MD<br>__Neuro/Psych<br>__SLP<br>__OT<br>__PT<br>__Rec<br>Other	Identify pharmacological treatments provided for sleep and duration of use.

**g. Behavioral Symptoms**				
16. Evaluate behavioral symptoms in accordance with existing EBTs.	Evaluate behavioral symptoms based on symptoms and guidelines (e.g., PCL or CAPS for PTSD)	NA	__MD<br>__Neuro/Psych<br>__SLP<br>__OT<br>__PT<br>__Rec<br>Other	Identify specific measures/approaches to evaluation of behavioral symptoms
**h. Cognitive Symptoms**				
17. For persons with cognitive complaints following non-responsiveness to treatment of comorbid conditions, refer for neuropsychological evaluation to assess functional limitations and guide treatment.	Refer for neuropsychological testing	NA	__MD<br>__Neuro/Psych<br>__SLP<br>__OT<br>__PT<br>__Rec<br>__Other	Identify referral to neuropsychological evaluation and specific measures used.
18. For persons with cognitive complaints following non-responsiveness to treatment of comorbid conditions, refer for cognitive rehabilitation	Refer for cognitive rehabilitation testing	NA	__MD<br>__Neuro/Psych<br>__SLP<br>__OT<br>__PT<br>__Rec<br>Other	Identify referral to cognitive rehabilitation and specific approaches used.
18a. For persons with cognitive complaints following non-responsiveness to treatment of comorbid conditions, provide short-term cognitive rehabilitation including strategy training and assistive technology/aids. Prolonged therapy is discouraged.	Provide short-term cognitive rehabilitation including strategy training and assistive technology/aids.	NA	__MD<br>__Neuro/Psych<br>__SLP<br>__OT<br>__PT<br>__Rec<br>__Other	Identify referral to cognitive rehabilitation, specific approaches used, and duration of treatment.
19. Do not use medications, supplements, nutraceuticals, or herbal medicines for ameliorating neurocognitive effects of mTBI.	NA	NA	__MD<br>__Neuro/Psych<br>__SLP<br>__OT<br>__PT<br>__Rec<br>__Other	Identify use of medications, supplements, nutraceuticals, or herbal medicines for ameliorating neurocognitive effects of mTBI.

D. Setting of Care				
20. Do not refer to specialty care for the majority of patients with mTBI.	N/A	N/A	N/A	N/A
21. For persons with persistent complaints following non-responsiveness to treatment of comorbid conditions, consult TBI specialty care for those with primary care driven refractory response to treatments.	Consult TBI Specialty Care	NA	__MD<br>__Neuro/Psych<br>__SLP<br>__OT<br>__PT<br>__Rec<br>__Other	Identify time from TBI to IETP to identify duration of symptoms.
22. For persons with persistent complaints following non-responsiveness to treatment of comorbid conditions, refer to case management within the primary care setting to provide.	Refer to case management within the primary care setting to provide.	N/A	__MD<br>__Neuro/Psych<br>__SLP<br>__OT<br>__PT<br>__Rec<br>__Other	Identify case management referral
23. Insufficient evidence to support interdisciplinary /multidisciplinary teams in management of patients with chronic symptoms.	N/A	N/A	N/A	N/A

Legend Denoting Disciplines: MD = Medical Doctor, Neuro/Psych = Neuropsychologist, SLP = Speech Language Pathologist, OT = Occupational Therapist, PT = Physical Therapist, Rec = Recreation Therapist.
