# Supplementary material for: Traumatic Brain Injury Intensive Evaluation and Treatment Program: Protocol for a Partnered Evaluation Initiative Mixed Methods Study
Source: JMIR Res Protoc. 2023 May 9;12:e44776. doi: 10.2196/44776 (PMC10206625; doi:10.2196/44776)
Supplement: Multimedia Appendix 3 [file resprot_v12i1e44776_app3.pdf]

Appendix 3

Aim 1

Veteran Demographic Questionnaire

Participant ID:__________________ **Interviewer:__________________________**

Date:____________________________

VETERAN AND SERVICE MEMBER DEMOGRAPHIC INFORMATION

OVERVIEW OF INJURY

1) What month and year were you diagnosed with TBI? ____________________________
2) Duration of Injury (in years): ____________________________
3) Mechanism of Injury: ____________________________
4) Admission Date into IETP program: ____________________________
5) Discharge Date from IETP program: ____________________________

COMMUNITY

6) Are you currently working (includes paying job and volunteer work)? (Circle One)

   Yes 1
   No 2

7) If yes, how many hours/week do you currently work? ____________________________
8) Where are you living now?

   Private home or apartment 1
   Communal facility, group home, or convalescent unit 2
   Hospital 3
   No fixed living arrangement 4
   Other, specify ______________________

DEMOGRAPHICS

9) What is your age in years? ____________________________
10) What is your gender? (Circle One)

   Male 1
   Female 2
   Other/prefer not to answer 3

Participant ID:____________________ **Interviewer:______________________________**

Date:_______________________________

11) What is your marital status? (Circle One)

Married/Civil Union 1
Separated 2
Divorced.. 3
Never Married 4
Partnered/not married... 5
Widowed 6

12) What category best represents your race/ethnicity? (Circle all that apply)

White..1
Black or African American 2
Asian..3
Native Hawaiian or Pacific Islander 4
American Indian or Alaska Native.................................5
Other, please specify ______________________

13) Are you of Hispanic, Latino, or Spanish origin?

No 1
Yes, Cuban 2
Yes, Puerto Rican... 3
Yes, Mexican, Mexican American, Chicano 4
Yes, another Hispanic, Latino, or Spanish origin,
please specify ______________________

14) What is the highest degree or level of school you have obtained? If you are currently enrolled mark the previous grade or highest degree received. (Circle one)

Less than 9th grade 1
9th through 12th grade, but did not finish high school 2
High School Graduate or GED 3
Some college but no degree 4
Associate degree 5
Bachelor's degree 6
Master's degree 7
Professional degree 8
Doctorate degree 9

MILITARY

15) What branch(es) of the service are or were you in?

Army 1
Navy 2
Air Force 3
Marines 4

16) What was your service status at the time of entry into TBI -RCM? (Circle One)

Active 1
Reserve 2
National Guard 3

17) What grade were you? (Circle one)

Enlisted 1
Officer 2

18) What is/was your job in the Military? ____

19) What were your dates of Service? ____

20) Did you deploy in support of the U.S. Armed Forces?______________________________

Participant ID:____________________ **Interviewer:______________________________**

Date:___________________________

	Deployed (Yes/ No)	How many times you deployed (Number)	Dates of Deployment(s) MM/YYYY-MM/YYYY	Did you serve in a combat zone* while deployed? (Yes/No)
Operation Iraqi Freedom (OIF)				
Operation Enduring Freedom (OEF)				
August 1990 to August 2001 (includes Persian Gulf War)				
May 1975 to July 1990				
Vietnam era (August 1964 to April 1975)				
February 1955 to July 1964				
Korean War (July 1950 to January 1955)				

Thank you!

Appendix 3
Aim 1
Focus Group Demographic Questionnaire

Characterization, Evaluation, and Implementation of Innovative TBI Intensive Evaluation and Treatment Programs (IETP)

Participant ID:____________________ **Interviewer:______________________________**

Date:_______________________________

PROVIDER DEMOGRAPHIC INFORMATION

EXPERIENCE

1) What is your current clinical position?
2) How long have you been in this position?
3) How many years' experience do you have working with persons with TBI?

DEMOGRAPHICS

4) What is your age in years?
5) What is your gender? (Circle One)

Male..1
Female..2
Other/prefer not to answer...3

6) What category best represents your race/ethnicity? (Circle all that apply)

White...1
Black or African American 2
Asian...3
Native Hawaiian or Pacific Islander 4
American Indian or Alaska Native..................................5
Other, please specify: ____________________________

Participant ID:____________________ **Interviewer:______________________________**

Date:______________________________

7) Are you of Hispanic, Latino, or Spanish origin?

No 1

Yes, Cuban 2

Yes, Puerto Rican… 3

Yes, Mexican, Mexican American, Chicano… 4

Yes, another Hispanic, Latino, or Spanish origin, please specify:

____

8) What is the highest degree or level of school you have obtained? If you are currently enrolled mark the previous grade or highest degree received. (Circle one)

Less than 9th grade 1

9th through 12th grade, but did not finish high school 2

High School Graduate or GED 3

Some college but no degree 4

Associate degree 5

Bachelor's degree... 6

Master's degree 7

Professional degree... 8

Doctorate degree 9

9) What professional licenses do you currently hold?

Registered Nurse.. 1
Advanced Practice Registered Nurse 2
Medical Doctor.. 3
Osteopathic Physician 4
Clinical Social Work... 5
Physical Therapist 6
Occupational Therapist.. 7
Orthotist 8
Speech-Language Pathologist 9
Audiologist 10
Dietician/ Nutritionist 11
Pharmacist... 12
Psychologist... 13
