# Supplementary material for: Traumatic Brain Injury Intensive Evaluation and Treatment Program: Protocol for a Partnered Evaluation Initiative Mixed Methods Study
Source: JMIR Res Protoc. 2023 May 9;12:e44776. doi: 10.2196/44776 (PMC10206625; doi:10.2196/44776)
Supplement: Multimedia Appendix 4 [file resprot_v12i1e44776_app4.pdf]

Appendix 4
Aim 1
Key Informant Interview Script

Characterization, Evaluation, and Implementation of Innovative TBI Intensive Evaluation and Treatment Program (TBI-IETP)

Participant ID: **Interviewer:**
Date: **Notetaker:**
Informant Role:

KEY INFORMANT INTERVIEW

OVERVIEW

Hello, my name is *[your name].*

Thank you for agreeing to participate in an interview for the "Characterization, Evaluation, and Implementation of Innovative TBI Intensive Evaluation and Treatment Program (TBI-IETP)" project. The TBI Intensive Evaluation and Treatment Program (IETP), is a new modality, or method, for delivering evidence-based care in a residential, inpatient format. IETP programs provide bundled evidence-based assessment, treatment, referral, and case management practices in concordance with existing guidelines for mild TBI and common co-occurring comorbidities (e.g., sleep disorders, chronic pain).The goal of the interview is to learn about [NAME OF LOCAL TBI-IETP program] at your site. I will ask you questions about (1) the core features of the program; (2) how the program is implemented; and (3) outcomes of the program. This information will help us understand the context in which this program is operating.

I am going to ask you open-ended questions about these topics. There are no right or wrong answers. I want to hear your thoughts so please do not hesitate to share.

We will audio-record this session to ensure accuracy in writing up our report. Your responses, however, will not be linked with your name. This interview is voluntary and has been approved by all VHA labor partners. Results will be presented anonymously and in aggregate.

Do you have any questions? *Answer any questions.*

With your permission, I would like to audio-record the interview.

Turn on the recorders, state your name, the date and time, your location, and participant ID.

Let's begin.

INTRODUCTION

1) Please describe your current position within the context of the [LOCAL PROGRAM]. (your role, how long at facility, how involved in the program) [*Characteristics of individuals*]

Characterization, Evaluation, and Implementation of Innovative TBI Intensive Evaluation and Treatment Program (TBI-IETP)

Participant ID: **Interviewer:**
Date: **Notetaker:**
Informant Role:

TBI-IETP PROGRAM AT YOUR SITE

We are interested in learning about your program.

2) I would like to understand the big picture perspective of your program. Tell me about your program. [*Intervention characteristics*]
    a. Please describe how your site's program began.
        i. When did it begin?
        ii. Why did this program start?
        iii. Who was involved in getting it started?
        iv. Who was the main person who made this program happen?
        v. At that time, what were the main goals for the program?
        vi. How have those goals changed?
        vii. To date, approximately how many patients have been served?

    b. Please describe the program as it current exists.
        i. Who involved with the program?
        ii. What does the program consist of?
            1. What are the activities/treatments? (duration of activities)
            2. How is your program delivered? (inpatient/outpatient; virtual; day treatment; residential, etc.)

    c. Please describe how patients become involved in the program.
        i. What are the requirements for patients to be admitted to the program? (who decides; what are the eligibility criteria)
        ii. Please describe the referral process.

    d. What does a patient's typical program experience look like?
        i. What do they experience?
        ii. How do you define patient success?
        iii. What contributes to a patient's success?

3) What are the ways you measure success of the program? (clinical outcomes; satisfaction; etc.) [*Implementation process*]

Characterization, Evaluation, and Implementation of Innovative TBI Intensive Evaluation and Treatment Program (TBI-IETP)

Participant ID: **Interviewer:**
Date: **Notetaker:**
Informant Role:

4) What supports the program's success? (space, staffing, leadership, location of service provision) [*Implementation process, Inner setting, Outer setting*]

5) What are some challenges the program has faced? [*Implementation process, Inner setting, Outer setting*]
    a. How have these challenges influenced the program?
    b. What are some ways these challenges have been addressed?

6) If you could change anything about the program, what would you change? [*Implementation process, Intervention characteristics*]
    a. Why those changes?
    b. What do you think would be the outcomes of those changes?

7) How has the program changed as a result of COVID-19? [*Inner setting, Outer setting*]
    a. What changes do you anticipate retaining long term?
    b. Why those changes?

INTERVIEW ACTIVITY

In an effort to operationalize your program components [TBI-IETP Care Implementation Elements Inventory] and your delivery model [Implementation Research Logic Model], we created a program inventory based on our knowledge of best practices.

8) Can you please review this with me and provide input about what is right or wrong and how it can be changed to better reflect your program?

NEXT STEPS

One of our next steps is to identify providers to participate in a focus group interview. The focus group will be scheduled at a time convenient for the participants and will be either virtual or in person depending on the situation.

Characterization, Evaluation, and Implementation of Innovative TBI Intensive Evaluation and Treatment Program (TBI-IETP)

Participant ID: **Interviewer:**
Date: **Notetaker:**
Informant Role:

9) Who do you recommend we contact at your site?
    Name: Position:
    a.

Additionally, we would like to invite you to participate in a follow-up interview. The follow up interview will discuss some processes associated with the program and other topics.

10) Would you be willing to have a follow up interview with us to validate data summaries and content developed as a result of the program evaluation?
    a. Yes b. No
