# Supplementary material for: Traumatic Brain Injury Intensive Evaluation and Treatment Program: Protocol for a Partnered Evaluation Initiative Mixed Methods Study
Source: JMIR Res Protoc. 2023 May 9;12:e44776. doi: 10.2196/44776 (PMC10206625; doi:10.2196/44776)
Supplement: Multimedia Appendix 5 [file resprot_v12i1e44776_app5.pdf]

Appendix 5

Aim 1

Site Visit Observation Guide and Comparison Checklist

Characterization, Evaluation, and Implementation of Innovative TBI Intensive Evaluation and Treatment Program (TBI-IETP)

Site ID: **Observers:**

Date:

OBSERVATION GUIDE - DRAFT

PHYSICAL CHARACTERISTICS

1) Describe where the PROGRAM is located at the site.

   a. Is it in one location or located in multiple areas?
   b. Are therapy rooms located together or in multiple areas?
   c. Are providers co-located or in multiple areas?
   d. Off site from main facility?

2) What resources/equipment are present? Describe

3) Residential/Inpatient: describe living arrangements

   a. Individual or shared rooms?

   b. Patient rooms located in same area or in different areas?

4) Outpatient Only

   a. When do they arrive/leave?

5) Other types of spaces: recreational, patient parking, social areas

6) Residential leave policies (for weekdays/weekends). Describe

7) What type of food is provided?

   a. Regular hospital food?

   b. Menu of customized food?

   c. Snacks provided?

   c. No food?

Characterization, Evaluation, and Implementation of Innovative TBI Intensive Evaluation and Treatment Program (TBI-IETP)

Site ID: **Observers:**

Date:

STAFFING AND COMMUNICATION

8) What types of providers are involved in the program? (circle all that apply)

a) Active Duty Military Liaison
b) Advanced Practice Nurse
c) Blind Rehabilitation Specialist
d) Certified Prosthetist/Orthotist
e) Chaplain
f) Dietician
g) Neuropsychologist
h) Nurse Case Manager
i) Occupational Therapist
j) Pharmacist
k) Physiatrist (rehabilitation doctor)
l) Physical Therapist
m) Recreation Therapist
n) Rehabilitation Nursing
o) Rehabilitation Psychologist
p) Social Work Case Manager
q) Speech and Language Pathologist
r) Vocational Rehabilitation Counselor
s) Other consultative services as needed (e.g., Neuro-ophthalmology, Orthopedics, Nutrition, Neurology, Psychiatry, Audiology, Surgery)

9) How do providers communicate with each other about patients?

a. IDT meetings, list other meetings, grand rounds, hallway conversations, consults, etc.

b. Who attends?

c. How often do they occur?

Characterization, Evaluation, and Implementation of Innovative TBI Intensive Evaluation and Treatment Program (TBI-IETP)

Site ID: **Observers:**

Date:

THERAPY

10) What therapies/services are provided? Describe.

    a. Adaptive sports
    b. Anger management
    c. Audiological rehabilitation
    d. Cognitive balance group
    e. Cognitive rehabilitation
    f. Community and family rehabilitation
    g. Headache treatment
    h. Individual prolonged exposure therapy for PTSD
    i. Insomnia and apnea treatment
    j. Medical care/Medical management
    k. Multi-sensory rehabilitation
    l. Pain management
    m. Physical therapy/Core training
    n. Relaxation training
    o. Vestibular therapy
    p. Vision therapy
    q. Vocational rehabilitation

11) Delivery models: individual, group, telehealth, and how it varies by type of therapy

Characterization, Evaluation, and Implementation of Innovative TBI Intensive Evaluation and Treatment Program (TBI-IETP)

Site ID: **Observers:**

Date:

LEADERSHIP

12) Who is considered leadership? Titles.

a) How are they involved?

SITE OBSERVATION COMPARISON

	Tampa	Minneapolis	Palo Alto	Richmond	San Antonio	Comments
PHYSICAL CHARACTERISTICS						
1. Where is the program located?						
a. In one location						
b. located in multiple areas						
c. Are providers co-located						
d. Off site from main facility						
2. What resources/equipment are present?						
a. (add)						
3. Residential/Inpatient: describe living arrangem ents						
a. Individual patient rooms						
b. Shared patient rooms						
c. Patient rooms in same area						
4. Outpatient only						

Characterization, Evaluation, and Implementation of Innovative TBI Intensive Evaluation and Treatment Program (TBI-IETP)

Site ID: **Observers:**

Date:

	Tampa	Minneapolis	Palo Alto	Richmond	San Antonio	Comments
a. When arrive/leave?						
5. Other types of spaces (add list)						
a. Designated patient parking						
b. Recreational space on site						
c. Social areas on site						
6. Residential leave policies on weekends						
a. (add)						
b. (add)						
7. Type of food served to patients						
a. Regular hospital food						
b. Menu of customized food						
c. (add)						
STAFFING AND COMMUNICATION						
8. What types of providers are involved in the program						
a. Physiatrist						
b. Social Work Case Manager						
c. Nurse Case Manager						
d. Physical Therapist						
e. Occupational Therapist						
f. Recreation Therapist						
g. Rehabilitation Nursing						
h. Advanced Practice Nurse						
i. Speech and Language Pathologist						
j. Rehabilitation Psychologist						

Characterization, Evaluation, and Implementation of Innovative TBI Intensive Evaluation and Treatment Program (TBI-IETP)

Site ID: **Observers:**

Date:

	Tampa	Minneapolis	Palo Alto	Richmond	San Antonio	Comments
k. Neuropsychologist						
l. Vocational Rehabilitation Counselor						
m. Active Duty Military Liaison						
n. Blind Rehabilitation Specialist						
o. Certified Prosthetist/Orthotist						
p. Chaplain						
q. Dietician						
r. Pharmacist						
s. Other consultative services as needed						
9. How do providers communicate with each other about patients?						
a. IDT meetings, list other meetings, grand rounds, hallway conversations, consults, etc.						
b. Who attends						
c. How often do they occur						
THERAPY						
10. What therapies/services are provided? (add list below)						
a. Adaptive sports						
b. Anger management						
c. Audiological rehabilitation						
d. Cognitive balance group						
e. Cognitive rehabilitation						
f. Community and family rehabilitation						
g. Headache treatment						

Characterization, Evaluation, and Implementation of Innovative TBI Intensive Evaluation and Treatment Program (TBI-IETP)

Site ID: **Observers:**

Date:

	Tampa	Minneapolis	Palo Alto	Richmond	San Antonio	Comments
h. Individual prolonged exposure therapy for PTSD						
i. Insomnia and apnea treatment						
j. Medical care/Medical management						
k. Multi-sensory rehabilitation						
l. Pain management						
m. Physical therapy/Core training						
n. Relaxation training						
o. Vestibular therapy						
p. Vision therapy						
q. Vocational rehabilitation						
r. (add)						
11. Delivery models (add by type of therapy)						
a. Individual						
b. Group						
c. Telehealth						
LEADERSHIP						
12. Who is considered leadership? (add list)						
a. How are they involved?						
