# Supplementary material for: Traumatic Brain Injury Intensive Evaluation and Treatment Program: Protocol for a Partnered Evaluation Initiative Mixed Methods Study
Source: JMIR Res Protoc. 2023 May 9;12:e44776. doi: 10.2196/44776 (PMC10206625; doi:10.2196/44776)
Supplement: Multimedia Appendix 6 [file resprot_v12i1e44776_app6.pdf]

Appendix 6
Aim 1
Focus Group Script

Characterization, Evaluation, and Implementation of Innovative TBI Intensive Evaluation and Treatment Program (TBI-IETP)

Focus Group ID:
Date:
Type of Focus Group:

Moderator:
Notetaker:
# of Participants:

FOCUS GROUP INTERVIEW

OVERVIEW

Hello, my name is *[your name].*

Thank you for agreeing to participate in an interview for the "Characterization, Evaluation, and Implementation of Innovative TBI Intensive Evaluation and Treatment Program (TBI-IETP)" project. The TBI Intensive Evaluation and Treatment Program (IETP), is a new modality, or method, for delivering evidence-based care in a residential, inpatient format. IETP programs provide bundled evidence-based assessment, treatment, referral, and case management practices in concordance with existing guidelines for mild TBI and common co-occurring comorbidities (e.g., sleep disorders, chronic pain).The goal of this focus group is to learn about the [NAME OF LOCAL PROGRAM] at your site. We will ask you questions about (1) the core features of the program; (2) how the program is implemented; and (3) outcomes that define success. This information will help us understand the context in which this program is operating.

We will ask open-ended questions about these topics. There are no right or wrong answers. We want to hear your thoughts so please do not hesitate to share.

We will audio-record this session to ensure accuracy in writing up our report. Your responses, however, will not be linked with your name. This session is voluntary and has been approved by all VHA labor partners. Results will be presented anonymously and in aggregate.

Do you have any questions? *Answer any questions.*

With your permission, we would like to audio-record the interview.

Turn on the recorders, state your name, the date and time, your location, and focus group ID.

Let's begin.

INTRODUCTION

1) First let's introduce ourselves by stating our first names and how you engage with the program. [*Characteristics of individuals*]

Characterization, Evaluation, and Implementation of Innovative TBI Intensive Evaluation and Treatment Program (TBI-IETP)

Focus Group ID: **Moderator:**
Date: **Notetaker:**
Type of Focus Group: **# of Participants:**

PROGRAM DESCRIPTION

We are interested in learning about your program.

2) If you wanted to tell another VA facility about your program, how would you describe it? [*Intervention characteristics*]
    a. What would they need to know to replicate your program at their facility?
        i. What makes your program unique?
    b. How are patients referred to the program?
        i. What are the requirements for patients to be admitted to the program? (who decides; what are the criteria; characteristics of patients in the program – age range, race, TBI severity and other comorbidities)

3) What is needed to successfully deliver the program? (staffing, facilities, equipment, materials, etc.) [*Implementation process*]

4) If you could change anything about the program, what would you change? [*Intervention characteristics*]
    a. Why those changes?
    b. What do you think would be the outcomes of those changes?

5) What are the ways you measure success of the program? [*Implementation process, Intervention characteristics*]
    a. What are short term outcomes?
    b. What are long term outcomes?

6) What supports the program's success? (space, staffing, leadership, location of service provision) [*Implementation process, Inner setting, Outer setting*]

7) What are some challenges the program has faced? [*Implementation process, Inner setting, Outer setting*]
    a. How have these challenges influenced the program?
    b. What are some ways these barriers/challenges have been addressed?

Characterization, Evaluation, and Implementation of Innovative TBI Intensive Evaluation and Treatment Program (TBI-IETP)

Focus Group ID: **Moderator:**
Date: **Notetaker:**
Type of Focus Group: **# of Participants:**

8) How has the program changed as a result of COVID-19? [*Inner setting, Outer setting*]
    a. What changes do you anticipate retaining long term? Why those changes?

FOCUS GROUP ACTIVITY

In an effort to operationalize your program components [TBI-IETP Care Implementation Elements Inventory] and your delivery model [Implementation Research Logic Model], we created a program inventory based on our knowledge of best practices.

9) Can you please review this with me and provide input about what is right or wrong and how it can be changed to better reflect your program?

CONCLUSION

10) Is there anything else you would like to add about your program before we conclude today's discussion?

We would like to invite you to participate in a follow-up interview. The follow up interview will discuss some processes associated with the program and other topics.

11) Would you be willing to have a follow up interview with us to validate data summaries and content developed as a result of the program evaluation?
    a. Yes (indicate who):

    b. No (indicate who):
