# Supplementary material for: Traumatic Brain Injury Intensive Evaluation and Treatment Program: Protocol for a Partnered Evaluation Initiative Mixed Methods Study
Source: JMIR Res Protoc. 2023 May 9;12:e44776. doi: 10.2196/44776 (PMC10206625; doi:10.2196/44776)
Supplement: Multimedia Appendix 7 [file resprot_v12i1e44776_app7.pdf]

Appendix 7

Aim 1

Follow-up Interview Script

Characterization, Evaluation, and Implementation of Innovative TBI Intensive Evaluation and Treatment Program (TBI-IETP)

Participant ID: **Interviewer:**
Date: **Notetaker:**
Informant Role:

FOLLOW-UP INTERVIEW

OVERVIEW

Hello, my name is *[your name].*

Thank you for agreeing to participate in an interview for the "Characterization, Evaluation, and Implementation of Innovative TBI Intensive Evaluation and Treatment Program (TBI-IETP)" project. The goal of the follow-up interview is to (1) review and discuss what we have learned about how your program is implemented and what is needed to be successful; and (2) obtain additional input and clarification to ensure accuracy.

I am going to ask you open-ended questions about these topics. There are no right or wrong answers. I want to hear your thoughts so please do not hesitate to share.

We will audio-record this session to ensure accuracy in writing up our report. Your responses, however, will not be linked with your name.

Do you have any questions? *Answer any questions.*

With your permission, I would like to audio-record the interview.

Turn on the recorders, state your name, the date and time, your location, and participant ID.

Let's begin.

INTRODUCTION

Let's start by having you introduce yourself,

1) Please describe your current position and role in the program. [*Characteristics of individuals*]

REVIEW OF PRELIMINARY FINDINGS

We conducted some individual interviews and focus group interviews at your site. From these data collection activities, I want to share some of the main preliminary findings to get your input on what we have learned and what we may be missing.

Present the main themes from the interviews and focus group interviews.

Characterization, Evaluation, and Implementation of Innovative TBI Intensive Evaluation and Treatment Program (TBI-IETP)

Participant ID: **Interviewer:**
Date: **Notetaker:**
Informant Role:

Main components of the program

2) Do you disagree/agree with what we found?

3) What would you add or change?

Present identified challenges

4) Do you disagree/agree with what we found?

5) What would you add or change?

Present identified supports

6) Do you disagree/agree with what we found?

7) What would you add or change?

Present other themes at emerged from this site

8) Do you disagree/agree with what we found?

9) What would you add or change?

CLARIFICATION OF FINDINGS

In our preliminary analyses, we realized that we need more information about [INSERT] to make sure we fully understand the program and how it is implemented.

Explain what we found about [INSERT] and ask follow-up questions such as:

10) What other information can you provide to help explain [INSERT]?

11) Can you provide an example of [INSERT]?

Characterization, Evaluation, and Implementation of Innovative TBI Intensive Evaluation and Treatment Program (TBI-IETP)

Participant ID: **Interviewer:**
Date: **Notetaker:**
Informant Role:

INTERVIEW ACTIVITY

As you may recall, as a part of the program evaluation, we developed the TBI-IETP Care Implementation Elements Inventory and delivery care models [Implementation Research Logic Model], and other relevant materials (e.g. data summaries, implementation checklists (e.g. staffing plan, program core aspects, impacts, etc.), educational content and templates). Could you please review these materials with me for relevance, accuracy, and usefulness?

Present material X (and repeat for each material)

12) Does this accurately reflect your program? [RELEVANCE]

13) Does this represent an ideal program from your perspective?

14) Are there things indicated that you do not do but would like to do in the future?

15) How would you revise it to better reflect your program? [ACCURACY]

16) Do you think [material X], would be useful in helping another facility implement a TBI rehabilitation and comorbidity management program? [USEFULNESS]

CONCLUSION

17) What would you change if you had to start all over again in implementing this program? Why?
