# Supplementary material for: Traumatic Brain Injury Intensive Evaluation and Treatment Program: Protocol for a Partnered Evaluation Initiative Mixed Methods Study
Source: JMIR Res Protoc. 2023 May 9;12:e44776. doi: 10.2196/44776 (PMC10206625; doi:10.2196/44776)
Supplement: Multimedia Appendix 8 [file resprot_v12i1e44776_app8.pdf]

Appendix 8
Aim 1
Veteran and
Service Member Interview
Script

Characterization, Evaluation, and Implementation of Innovative TBI Intensive Evaluation and Treatment Program (TBI-IETP)

Participant ID: **Interviewer:**
Date: **Notetaker:**

VETERAN INTERVIEW

OVERVIEW

Hello, my name is *[your name].*

Thank you for agreeing to participate in an interview for the “Characterization, Evaluation, and Implementation of Innovative TBI Intensive Evaluation and Treatment Program (TBI-IETP)” project. The TBI Intensive Evaluation and Treatment Program (IETP), is a new modality, or method, for delivering evidence-based care in a residential, inpatient format. IETP programs provide bundled evidence-based assessment, treatment, referral, and case management practices in concordance with existing guidelines for mild TBI and common co-occurring comorbidities (e.g., sleep disorders, chronic pain).The goal of our interview is to learn about your experiences with the [NAME OF LOCAL PROGRAM]. I will ask you questions about (1) the services you received; and (2) outcomes that you experienced. This information will help us understand the context in which this program is operating.

I am going to ask you open-ended questions about these topics. There are no right or wrong answers. I want to hear your thoughts so please do not hesitate to share.

We will audio-record this session to ensure accuracy in writing up our report. Your responses, however, will not be linked with your name. Your participation in this project is anonymous, voluntary and will not affect your care or benefits at the VA. You can stop participating at any time.

Do you have any questions? *Answer any questions.*

With your permission, I would like to audio-record the interview.

Turn on the recorders, state your name, the date and time, your location, and ID.

Let’s begin.

VETERAN PROGRAM EXPERIENCES

We want to understand your experiences with NAME OF PROGRAM to better service other veterans like you in the future.

1) How did you learn about the program? [*Implementation process*]

2) If you wanted to tell another Veteran about the program, how would you describe it? [*Intervention characteristics*]

Participant ID: **Interviewer:**
Date: **Notetaker:**

3) What made you want to participate in the program? [*Characteristics of individuals, Intervention characteristics, Implementation process*]

4) Please tell me about your experiences in the program. [*Intervention characteristics*]
    a. What kind of services did you receive?
    b. What services were most helpful?
    c. Describe services that you wished you received.

5) What are some challenges you faced when in the program? [*Characteristics of individuals, Intervention characteristics*]
    a. How have these challenges influenced your involvement?
    b. What are some ways these challenges have been addressed?

6) What helped you the most when in the program? [*Characteristics of individuals, Intervention characteristics*]

7) How did this program affect your life/functioning? [*Characteristics of individuals, Intervention characteristics, Implementation process*]
    a. Work
    b. Relationship
    c. Health (physical, mental, emotional, cognitive)

8) If you could change anything about the program, what would you change? [*Intervention characteristics*]

CONCLUSION

9) Is there anything else you would like to add about your program before we conclude today’s discussion?
