# Supplementary material for: Traumatic Brain Injury Intensive Evaluation and Treatment Program: Protocol for a Partnered Evaluation Initiative Mixed Methods Study
Source: JMIR Res Protoc. 2023 May 9;12:e44776. doi: 10.2196/44776 (PMC10206625; doi:10.2196/44776)
Supplement: Multimedia Appendix 9 [file resprot_v12i1e44776_app9.pdf]

Appendix 9

Aim 1

DoD Stakeholder Interview Script

Characterization, Evaluation, and Implementation of Innovative TBI Intensive Evaluation and Treatment Program (TBI-IETP)

Participant ID:
Date:
Informant Role:
Interviewer:
Notetaker:

DEPARTMENT OF DEFENSE STAKEHOLDER INTERVIEW

OVERVIEW

Hello, my name is *[your name].*

Thank you for agreeing to participate in an interview for the "Characterization, Evaluation, and Implementation of Innovative TBI Intensive Evaluation and Treatment Program (TBI-IETP)" project. The TBI Intensive Evaluation and Treatment Program (IETP), is a new modality, or method, for delivering evidence-based care in a residential, inpatient format. IETP programs provide bundled evidence-based assessment, treatment, referral, and case management practices in concordance with existing guidelines for mild TBI and common co-occurring comorbidities (e.g., sleep disorders, chronic pain).The goal of this interview is to (1) discuss what your experiences with the programs have been; and, (2) what works well or could use improvement.

I am going to ask you open-ended questions about these topics. There are no right or wrong answers. I want to hear your thoughts so please do not hesitate to share.

We will audio-record this session to ensure accuracy in writing up our report. Your responses, however, will not be linked with your name. This interview is voluntary and has been approved by all VHA labor partners. Results will be presented anonymously and in aggregate.

Do you have any questions? *Answer any questions.*

With your permission, I would like to audio-record the interview.

Turn on the recorders, state your name, the date and time, your location, and participant ID.

Let's begin.

INTRODUCTION

Let's start by having you introduce yourself,

1) Please describe your current position and role in the program. (your role, how involved in the program) [*Characteristics of individuals*]

Characterization, Evaluation, and Implementation of Innovative TBI Intensive Evaluation and Treatment Program (TBI-IETP)

Participant ID: **Interviewer:**
Date: **Notetaker:**
Informant Role:

PROGRAM EXPERIENCES

We are interested in learning about active duty service members in the program.

1) How are active duty service members identified for the program? [*Intervention characteristics, Implementation process*]
   a. What criteria?
   b. What makes a good candidate?

2) What do you expect to happen when you refer an active duty service member to the program? [*Implementation process*]
   a. What is your role in the referral process?
   b. What makes it an easy process?
   c. What makes it a challenging process?

3) Once an active duty service member is enrolled, what is the best-case scenario for that person? [*Intervention characteristics, Characteristics of individuals*]
   a. What services are provided?
   b. What information is provided to you during this time?
   c. What is your role during this time?

4) What works well with the program? [*Characteristics of individuals, Intervention characteristics, Implementation process*]
   a. What is “value-added” about the program?
   b. What is missing? What do you wish were part of the program?

5) Please provide an example when a participant’s experience didn’t go well. [*Intervention characteristics, Implementation process*]
   a. How did you learn about it?
   b. What information did you receive?
   c. What did you do?

6) What are some outcomes you expect to occur from the active duty service members who participate in the program? [*Implementation process*]

Characterization, Evaluation, and Implementation of Innovative TBI Intensive Evaluation and Treatment Program (TBI-IETP)

Participant ID:
Date:
Informant Role:
Interviewer:
Notetaker:

7) What are the ways you measure success of the program? (clinical outcomes; satisfaction; etc.) [*Implementation process*]

8) How has the program changed as a result of COVID-19? [*Inner setting, Outer setting*]
    a. How did these changes impact your role with the program?
    b. What changes do you anticipate will remain?
    c. Why those changes?

9) In terms of the future, how long do you foresee referring active duty service members to this program? [*Implementation process, Inner setting*]

CONCLUSION

10) If you could change anything about the program, what would you change? [*Intervention characteristics*]
    a. Why those changes?
    b. What do you think would be the outcomes of those changes?

11) Is there anything else you would like to add about the program before we conclude today's discussion?
