# Supplementary material for: Traumatic Brain Injury Intensive Evaluation and Treatment Program: Protocol for a Partnered Evaluation Initiative Mixed Methods Study
Source: JMIR Res Protoc. 2023 May 9;12:e44776. doi: 10.2196/44776 (PMC10206625; doi:10.2196/44776)
Supplement: Multimedia Appendix 11 [file resprot_v12i1e44776_app11.pdf]

Appendix 11

Aim 1

Implementation Elements Inventory Example

Cognitive Rehab Treatment Factors

ORION Record ID ______________________

The fields in this form should be filled out at each encounter

Encounter ID ______________________

Provider Code ______________________

Provider's primary specialty/ discipline
- ◯ Clinical Neuropsychologist
- ◯ Occupational Therapist
- ◯ Speech-Language Pathologist

Was the appointment telehealth?
- ◯ Yes
- ◯ No

Was this appointment kept?
- ◯ Yes
- ◯ No

Not kept because of
- ◯ No show
- ◯ Facility cancel
- ◯ Patient cancel
- ◯ Admin cancel

Was a clinical support staff member utilized in this encounter?
- ◯ Yes
- ◯ No

Type of clinical support staff member was
- ◯ Occupational Therapy Assistant
- ◯ Behavioral Health Technician (Psychometrist)
- ◯ Behavioral Health Technician (Neuro-Psychometrist)
- ◯ Behavioral Health Technician
- ◯ Recreational Therapist

Clinical support staff member code ______________________

Access to care: Internal to ISC (Specific to the discipline)

Please provide number in days from Program enrollment to initial discipline specific appointment ______________________

Alternating Attention

Tool/Mode of Delivery the provider used for Alternating Attention treatment	☐ Education<br>☐ Practical Exercise<br>☐ Activity<br>☐ Tool, Technology<br>☐ Tool, Non-Technology<br>☐ Complementary Alternative MED-CAM (Referral and Tracking only)
Education tools the provider used for Alternating Attention treatment	☐ Verbal<br>☐ Reading/handouts<br>☐ Video
Practical Exercise tools the provider used for Alternating Attention treatment	☐ In session demonstration<br>☐ In session distributed practice<br>☐ In session intensive practice<br>☐ Homework
Activity tools the provider used for Alternating Attention treatment	☐ Cognitive Stimulation<br>☐ Sensory Stimulation<br>☐ Cognitive Remediation (Restorative) activities/drills<br>☐ Compensation training, Internal - Active listening<br>☐ Compensation training, Internal - Restating<br>☐ Compensation training, Internal - Slowing Down<br>☐ Compensation training, Internal - Other<br>☐ Compensatory training, external<br>☐ Formalized Training Program: Strategic Memory and Reasoning Training (SMART)<br>☐ Formalized Training Program: Compensatory Cognitive Training (CCT)/CogSMART<br>☐ Formalized Training Program: Short Term Executive Plus (STEP)<br>☐ Formalized Training Program: Goal Management Training<br>☐ Problem Solving Therapy
Technology tools the provider used for Alternating Attention treatment	☐ Smartphone, calendar<br>☐ Smart Pen<br>☐ Mobile apps<br>☐ Video game<br>☐ Video game & exercise (Brain Bike)<br>☐ Audio Recording<br>☐ Alpha Stim<br>☐ Auricular Acupuncture<br>☐ TENS<br>☐ Attention Processing Test<br>☐ Other
Non-Technology tools the provider used for Alternating Attention treatment	☐ External, written cognitive aids (e.g., note taking, post-it reminders)<br>☐ External cognitive aids (visual cues)<br>☐ External cognitive aids (auditory cues - simple alarms)<br>☐ Environmental modification (e.g., reduce distractions)<br>☐ Journaling (paper)<br>☐ Repeat measure charts (e.g., BDI pattern)<br>☐ Written practical exercises<br>☐ Neuropsychometric tool/outcomes

Fear due to anxiety or trauma

Tool/Mode of Delivery the provider used for Fear due to anxiety or trauma treatment	☐ Education<br>☐ Practical Exercise<br>☐ Pharmacologic<br>☐ BH/Psychotherapy<br>☐ Tool, Technology<br>☐ Tool, Non-Technology<br>☐ Complementary Alternative MED-CAM (Referral and Tracking only)
Education tools the provider used for Fear due to anxiety or trauma treatment	☐ Verbal<br>☐ Reading/handouts<br>☐ Video
Practical Exercise tools the provider used for Fear due to anxiety or trauma treatment	☐ In session demonstration<br>☐ In session distributed practice<br>☐ In session intensive practice<br>☐ Homework
Pharmacologic tools the provider used for Fear due to anxiety or trauma treatment	☐ OTC<br>☐ Herbal<br>☐ Prescription, oral
BH/Psychotherapy tools the provider used for Fear due to anxiety or trauma treatment	☐ Cognitive Restructuring<br>☐ Problem Solving Therapy<br>☐ Challenging Irrational Thoughts<br>☐ Relaxation Therapy/training<br>☐ Systematic Desensitization, with Relaxation<br>☐ Prolonged Exposure<br>☐ Interpersonal (Yalom) Group<br>☐ CBT-Insomnia<br>☐ EMDR<br>☐ Supportive Counseling<br>☐ Cognitive Processing Therapy<br>☐ Dialectic Behavior Therapy<br>☐ Social Skills training<br>☐ Paradoxical therapies<br>☐ Behavioral Modification<br>☐ Cognitive Behavioral Therapy<br>☐ Psychodynamic or Insight Oriented Therapy<br>☐ Humanistic/Existential Therapy
Technology tools the provider used for Fear due to anxiety or trauma treatment	☐ Smartphone, calendar log<br>☐ Biofeedback<br>☐ Mobile apps<br>☐ Video game<br>☐ Audio Recording<br>☐ Virtual exposure (VR)
Non-Technology tools the provider used for Fear due to anxiety or trauma treatment	☐ Imaginal exposure<br>☐ In vivo exposure (ex. blast sounds)<br>☐ Symptom Log (paper)<br>☐ Journaling (paper)<br>☐ Sleep mask<br>☐ Repeat measure charts (e.g., BDI pattern)
