# Supplementary material for: Traumatic Brain Injury Intensive Evaluation and Treatment Program: Protocol for a Partnered Evaluation Initiative Mixed Methods Study
Source: JMIR Res Protoc. 2023 May 9;12:e44776. doi: 10.2196/44776 (PMC10206625; doi:10.2196/44776)
Supplement: Multimedia Appendix 12 [file resprot_v12i1e44776_app12.pdf]

Appendix 12
Aim 2
IETP Chart Abstraction Overview

The items below are examples of chart abstraction tools from the ORION project. These and other components will be edited, adapted, and added based on collaboration with site and operational stakeholders and the fidelity assessment.

Behavioral Health Emotional Treatment Factors

STUDY ID	______________________

The fields in this form should be filled out at each encounter

Provider's primary specialty/ discipline	Clinical Social Worker<br>Clinical/Counseling Psychologist<br>Occupational Therapist
Any missed appointments?	◯ Yes<br>◯ No
Not kept because of	◯ No show<br>◯ Facility cancel<br>◯ Patient cancel<br>◯ Admin cancel

Access to care: Internal to ISC (Specific to the discipline)

Please provide number in days from Program enrollment to initial discipline specific appointment	______________________

Access to care for that appointment

Type of Session	◯ Individual<br>◯ Group
# of participants in the group session	______________________
Location	◯ Individual office setting<br>◯ Examination Room<br>◯ Outdoor activity space<br>◯ Education/Group Room
Characterization of environment	◯ Quiet<br>◯ Visually Distracting<br>◯ Auditory Distractions<br>◯ Active
Caregiver present	◯ Yes<br>◯ No
Caregiver Relationship	◯ Spouse<br>◯ Parent<br>◯ Sibling<br>◯ Friend
Command representative present	◯ Yes<br>◯ No
Patient validation	Provider acknowledges patient success<br>Provider acknowledges patient lack of success (challenge)

Compliance

Any indicators of non-compliance?	Yes

No
NA
(If no homework, please select NA)

Relationship

Evidence of difficulty with rapport in the note
Evidence of lack of command support in the note
Report of command non-compliance with profile
Documented provider consultation with command (not profile)
◯ Documented team member consultation with command

BH Treatment Factors	
Please select the target(s)/symptom(s) the patient came to the clinic struggling with	☐ Mood/Anxiety/Trauma<br>☐ Executive<br>☐ Social Cognition<br>☐ Sleep<br>☐ Pain

Mood/Anxiety/Trauma	
Mood/Anxiety/Trauma symptom(s) under treatment	☐ Depressive mood<br>☐ Inflated mood<br>☐ Anhedonia<br>☐ Mood Reactivity<br>☐ Anger/Irritability<br>☐ Avoidance due to anxiety or trauma<br>☐ Fear due to anxiety or trauma<br>☐ Physical reactivity resulting from anxiety or trauma<br>☐ Cognitive symptoms demonstrating anxiety (e.g., rumination)<br>☐ Distorted cognitions related to trauma (e.g., guilt, shame, catastrophizing, etc.)<br>☐ Inability to recall an important event (in trauma, not due to LOC, AOC, or injury-specific PTA)<br>☐ Recurrent, intrusive recall (flashbacks)<br>☐ Somatization

Approaches for any documented BH issues identified	
Tool/Mode of Delivery the provider used for the **BH issue** treatment	☐ Education<br>☐ Practical Exercise<br>☐ Pharmacologic<br>☐ BH/Psychotherapy<br>☐ Tool, Technology<br>☐ Tool, Non-Technology<br>☐ Complementary Alternative MED-CAM (Referral and Tracking only)
Education tools the provider used for **BH issue** treatment	☐ Verbal<br>☐ Reading/handouts<br>☐ Video
Practical Exercise tools the provider used for **BH issue** treatment	☐ In session demonstration<br>☐ In session distributed practice<br>☐ In session intensive practice<br>☐ Homework
Pharmacologic tools the provider used for **BH issue** treatment	☐ OTC<br>☐ Herbal<br>☐ Prescription, oral

BH/Psychotherapy tools the provider used for **BH issue** treatment	☐ Cognitive Restructuring<br>☐ Problem Solving Therapy<br>☐ Challenging Irrational Thoughts<br>☐ Relaxation Therapy/training<br>☐ Systematic Desensitization, with Relaxation<br>☐ Prolonged Exposure<br>☐ Interpersonal (Yalom) Group<br>☐ CBT-Insomnia<br>☐ EMDR<br>☐ Supportive Counseling<br>☐ Cognitive Processing Therapy<br>☐ Dialectic Behavior Therapy<br>☐ Social Skills training<br>☐ Paradoxical therapies<br>☐ Behavioral Modification<br>☐ Cognitive Behavioral Therapy<br>☐ Psychodynamic or Insight Oriented Therapy<br>☐ Humanistic/Existential Therapy
Technology tools the provider used for **BH issue** treatment	☐ Smartphone, calendar log<br>☐ Biofeedback<br>☐ Mobile apps<br>☐ Video game<br>☐ Audio Recording<br>☐ Virtual exposure (VR)
Non-Technology tools the provider used for **BH issue** treatment	☐ Imaginal exposure<br>☐ In vivo exposure (ex. blast sounds)<br>☐ Symptom Log (paper)<br>☐ Journaling (paper)<br>☐ Sleep mask<br>☐ Repeat measure charts (e.g., BDI pattern)
Complementary Alternative MED-CAM tools the provider used for **BH issue** treatment	☐ Art Therapy<br>☐ Aquatics<br>☐ Archery<br>☐ Chiropractic Medicine<br>☐ Equine Therapy<br>☐ Massage<br>☐ Meditation<br>☐ Tai Chi<br>☐ Yoga<br>☐ Qigong<br>☐ Aromatherapy

Cognitive Rehab Treatment Factors

STUDY ID ______________________

The fields in this form should be filled out at each encounter

Provider's primary specialty/ discipline
- ◯ Clinical Neuropsychologist
- ◯ Occupational Therapist
- ◯ Speech-Language Pathologist

Access to care: Internal to ISC (Specific to the discipline)

Please provide number in days from Program enrollment to initial discipline specific appointment ______________________

Access to care for that appointment

Time of Encounter

Type of Session
- ◯ Individual
- ◯ Group

# of participants in the group session ______________________

Location
- ◯ Individual office setting
- ◯ Examination Room
- ◯ Outdoor activity space
- ◯ Education/Group Room

Caregiver present
- ◯ Yes
- ◯ No

Caregiver Relationship
- ◯ Spouse
- ◯ Parent
- ◯ Sibling
- ◯ Friend

Command representative present
- ◯ Yes
- ◯ No

Patient validation
- ◯ Provider acknowledges patient success
- ◯ Provider acknowledges patient lack of success (challenge)

Compliance

Notation of patient compliance/noncompliance
- ◯ Yes
- ◯ No
- ◯ NA

(If no homework, please select NA)

Relationship
- ◯ Evidence of difficulty with rapport in the note
- ◯ Evidence of lack of command support in the note
- ◯ Report of command non-compliance with profile
- ◯ Documented provider consultation with command (not profile)
- ◯ Documented team member consultation with command

SLP and Neuropsych Treatment Factors	
Please select the target(s)/symptom(s) the patient came to the clinic struggling with	☐ Attention<br>☐ Memory<br>☐ Executive<br>☐ Social Cognition<br>☐ Language

Attention	
Attention symptom(s) under treatment	☐ Sustained Attention<br>☐ Focused Attention<br>☐ Alternating Attention<br>☐ Divided Attention<br>☐ Attention, Not otherwise defined

These items will be identified for each attention symptom noted above	
Tools/Modes of Delivery the provider used for ATTENTION SYMPTOM treatment	☐ Education<br>☐ Practical Exercise<br>☐ Activity<br>☐ Tool, Technology<br>☐ Tool, Non-Technology<br>☐ Complementary Alternative MED-CAM (Referral and Tracking only)
Education tools the provider used for ATTENTION SYMPTOM treatment	☐ Verbal<br>☐ Reading/handouts<br>☐ Video
Practical Exercise tools the provider used for ATTENTION SYMPTOM treatment	☐ In session demonstration<br>☐ In session distributed practice<br>☐ In session intensive practice<br>☐ Homework
Activity tools the provider used for ATTENTION SYMPTOM treatment	☐ Cognitive Stimulation<br>☐ Sensory Stimulation<br>☐ Cognitive Remediation (Restorative) activities/drills<br>☐ Compensation training, Internal - Active listening<br>☐ Compensation training, Internal - Restating<br>☐ Compensation training, Internal - Slowing Down<br>☐ Compensation training, Internal - Other<br>☐ Compensatory training, external<br>☐ Formalized Training Program: Strategic Memory and Reasoning Training (SMART)<br>☐ Formalized Training Program: Compensatory Cognitive Training (CCT)/CogSMART<br>☐ Formalized Training Program: Short Term Executive Plus (STEP)<br>☐ Formalized Training Program: Goal Management Training<br>☐ Problem Solving Therapy

Technology tools the provider used for ATTENTION SYMPTOM treatment	☐ Smartphone, calendar<br>☐ Smart Pen<br>☐ Video game<br>☐ Video game & exercise (Brain Bike)<br>☐ Audio Recording<br>☐ Attention Processing Test (APT)<br>☐ Interactive Metronome (IM)<br>☐ Other computerized intervention (therapist-guided)<br>☐ Other computerized/app driven intervention (self-guided)<br>☐ Neuropsychometric tool/outcomes
Non-Technology tools the provider used for ATTENTION SYMPTOM treatment	☐ External, written cognitive aids (e.g., note taking, post-it reminders)<br>☐ External cognitive aids (visual cues)<br>☐ External cognitive aids (auditory cues - simple alarms)<br>☐ Environmental modification (e.g., reduce distractions)<br>☐ Journaling (paper)<br>☐ Repeat measure charts (e.g., BDI pattern)<br>☐ Written practical exercises<br>☐ Neuropsychometric tool/outcomes
Complementary Alternative MED-CAM tools the provider used for ATTENTION SYMPTOM treatment	☐ Art Therapy<br>☐ Aquatics<br>☐ Archery<br>☐ Chiropractic Medicine<br>☐ Equine Therapy<br>☐ Massage<br>☐ Meditation<br>☐ Tai Chi<br>☐ Yoga<br>☐ Qigong<br>☐ Aromatherapy
