# Supplementary material for: Traumatic Brain Injury Intensive Evaluation and Treatment Program: Protocol for a Partnered Evaluation Initiative Mixed Methods Study
Source: JMIR Res Protoc. 2023 May 9;12:e44776. doi: 10.2196/44776 (PMC10206625; doi:10.2196/44776)
Supplement: Multimedia Appendix 13 [file resprot_v12i1e44776_app13.pdf]

Appendix 13
Aim 2
Long-term Outcomes Survey

Mayo-Portland Adaptability Inventory-4

Participation Index (M2PI)

Muriel D. Lezak, PhD, ABPP & James F. Malec, PhD, ABPP

Name: ____ Clinic # ________________________ Date _______________

Person reporting (circle one): Single Professional Professional Consensus Person with brain injury Significant other: ________

Below each item, circle the number that best describes the level at which the person being evaluated experiences problems. Mark the greatest level of problem that is appropriate. Problems that interfere rarely with daily or valued activities, that is, less than 5% of the time, should be considered not to interfere. Write comments about specific items at the end of the rating scale.

**1. Initiation:** Problems getting started on activities without prompting				
**0** None	**1** Mild problem but does <u>not</u> interfere with activities; may use assistive device or medication	**2** Mild problem; interferes with activities 5-24% of the time	**3** Moderate problem; interferes with activities 25-75% of the time	**4** Severe problem; interferes with activities more than 75% of the time
**2. Social contact with friends, work associates, and other people who are not family, significant others, or professionals**				
**0** Normal involvement with others	**1** Mild difficulty in social situations but maintains normal involvement with others	**2** Mildly limited involvement with others (75-95% of normal interaction for age)	**3** Moderately limited involvement with others (25-74% of normal interaction for age)	**4** No or rare involvement with others (less than 25% of normal interaction for age)
**3. Leisure and recreational activities**				
**0** Normal participation in leisure activities for age	**1** Mild difficulty in these activities but maintains normal participation	**2** Mildly limited participation (75-95% of normal participation for age)	**3** Moderately limited participation (25-74% of normal participation for age)	**4** No or rare participation (less than 25% of normal participation for age)
4. **Self-care:** Eating, dressing, bathing, hygiene				
**0** Independent completion of self-care activities	**1** Mild difficulty, occasional omissions or mildly slowed completion of self-care; may use assistive device or require occasional prompting	**2** Requires a little assistance or supervision from others (5-24% of the time) including frequent prompting	**3** Requires moderate assistance or supervision from others (25-75% of the time)	**4** Requires extensive assistance or supervision from others (more than 75% of the time)
5. Residence: Responsibilities of independent living and homemaking(such as meal preparation, home repairs and maintenance, personal health maintenance beyond basic hygiene including medical management) but not including managing money (see # 8)				
**0** Independent; living without supervision or concern from others	**1** Living without supervision but others have concerns about safety or managing responsibilities	**2** Requires a little assistance or supervision from others (5-24% of the time)	**3** Requires moderate assistance or supervision from others (25-75% of the time)	**4** Requires extensive assistance or supervision from others (more than 75% of the time)
**6. *Transportation**				
**0** Independent in all modes of transportation including independent ability to operate a personal motor vehicle	**1** Independent in all modes of transportation, but others have concerns about safety	**2** Requires a little assistance or supervision from others (5-24% of the time); cannot drive	**3** Requires moderate assistance or supervision from others (25-75% of the time); cannot drive	**4** Requires extensive assistance or supervision from others (more than 75% of the time); cannot drive

DC 1YR 2YR

**7A. *Paid Employment:** Rate either item 7A or 7B to reflect the primary desired social role. Do not rate both. Rate 7A if the primary social role is paid employment. If another social role is primary, rate only 7B. For both 7A and 7B, "support" means special help from another person with responsibilities (such as, a job coach or shadow, tutor, helper) or reduced responsibilities. Modifications to the physical environment that facilitate employment are not considered as support.				
**0** Full-time (more than 30 hrs/wk) without support	**1** Part-time (3 to 30 hrs/ wk) without support	**2** Full-time or part-time with support	**3** Sheltered work	**4** Unemployed; employed less than 3 hours per week
**7B. *Other employment:** Involved in constructive, role-appropriate activity other than paid employment.<br>Check only one to indicate primary desired social role:<br>Childrearing/care-giving Homemaker, no childrearing or care-giving Student<br>Volunteer Retired (Check retired only if over age 60; if unemployed, retired as disabled and under age 60, indicate "Unemployed" for item 7A.				
**0** Full-time (more than 30 hrs/wk) without support; full-time course load for students	**1** Part-time (3 to 30 hrs/ wk) without support	**2** Full-time or part-time with support	**3** Activities in a supervised environment other than a sheltered workshop	**4** Inactive; involved in role-appropriate activities less than 3 hours per week
**8. Managing money and finances:** Shopping, keeping a check book or other bank account, managing personal income and investments				
**0** Independent, manages money without supervision or concern from others	**1** Manages money independently but others have concerns	**2** Requires mild assistance or supervision from others (5-24% of the time)	**3** Requires moderate assistance or supervision from others (25-75% of the time)	**4** Requires extensive assistance or supervision from others (more than 75% of the time)

Comments:

Item #

_____ ____

_____ ____

_____ ____

Items with an asterisk (6, 7A or 7B) require rescoring as specified below before raw scores are summed and referred to reference tables to obtain standard T-scores.

Rescore item 6. Original score = _____
If original score = 0 or 1, new score = 0
If original score = 2 or 3, new score = 1
If original score = 4, new score = 3

Rescore item 7A or 7B. Original score = _____
If original score = 0, new score = 0
If original score = 1 or 2, new score = 1
If original score = 3 or 4, new score = 3

New score for item 6 = _____
New score for item 7Aor 7B = _____
Sum of scores for other items (1-5 & 8) _____
TOTAL = _____ Standard T-score = ______

Name: ______________________

NEUROBEHAVIORAL SYMPTOM INVENTORY

Please rate the following symptoms with regard to how much they have disturbed you *SINCE YOUR INJURY*.

0 = None- Rarely if ever present; not a problem at all

1 = Mild- Occasionally present, but it does not disrupt activities; I can usually continue what I'm doing; doesn't really concern me.

2 = Moderate- Often present, occasionally disrupts my activities; I can usually continue what I'm doing with some effort; I feel somewhat concerned.

3 = Severe- Frequently present and disrupts activities; I can only do things that are fairly simple or take little effort; I feel like I need help.

4 = Very Severe- Almost always present and I have been unable to perform at work, school or home due to this problem; I probably cannot function without help.

1. Feeling dizzy:

0	1	2	3	4
NONE	MILD	MODERATE	SEVERE	VERY SEVERE

2. Loss of balance:

0	1	2	3	4
NONE	MILD	MODERATE	SEVERE	VERY SEVERE

3. Poor coordination, clumsy:

0	1	2	3	4
NONE	MILD	MODERATE	SEVERE	VERY SEVERE

4. Headaches:

0	1	2	3	4
NONE	MILD	MODERATE	SEVERE	VERY SEVERE

5. Nausea:

0	1	2	3	4
NONE	MILD	MODERATE	SEVERE	VERY SEVERE

6. Vision problems, blurring, trouble seeing:

0	1	2	3	4
NONE	MILD	MODERATE	SEVERE	VERY SEVERE

Name: ___________________________

7. Sensitivity to light

0	1	2	3	4
NONE	MILD	MODERATE	SEVERE	VERY SEVERE

8. Hearing difficulty:

0	1	2	3	4
NONE	MILD	MODERATE	SEVERE	VERY SEVERE

9. Sensitivity to noise:

0	1	2	3	4
NONE	MILD	MODERATE	SEVERE	VERY SEVERE

10. Numbness or tingling on parts of my body:

0	1	2	3	4
NONE	MILD	MODERATE	SEVERE	VERY SEVERE

11. Change in taste and/or smell:

0	1	2	3	4
NONE	MILD	MODERATE	SEVERE	VERY SEVERE

12. Loss of appetite or increase appetite:

0	1	2	3	4
NONE	MILD	MODERATE	SEVERE	VERY SEVERE

13. Poor concentration, can't pay attention, easily distracted:

0	1	2	3	4
NONE	MILD	MODERATE	SEVERE	VERY SEVERE

14. Forgetfulness, can't remember things:

0	1	2	3	4
NONE	MILD	MODERATE	SEVERE	VERY SEVERE

15. Difficulty making decisions:

0	1	2	3	4
NONE	MILD	MODERATE	SEVERE	VERY SEVERE

16. Slowed thinking, difficulty getting organized, can't finish things:

0	1	2	3	4
NONE	MILD	MODERATE	SEVERE	VERY SEVERE

17. Fatigue, loss of energy, getting tired easily:

0	1	2	3	4
NONE	MILD	MODERATE	SEVERE	VERY SEVERE

Name: ________________________

18. Difficulty falling or staying asleep:

0	1	2	3	4
NONE	MILD	MODERATE	SEVERE	VERY SEVERE

19. Feeling anxious or tense:

0	1	2	3	4
NONE	MILD	MODERATE	SEVERE	VERY SEVERE

20. Feeling depressed or sad:

0	1	2	3	4
NONE	MILD	MODERATE	SEVERE	VERY SEVERE

21. Irritability, easily annoyed:

0	1	2	3	4
NONE	MILD	MODERATE	SEVERE	VERY SEVERE

22. Poor frustration tolerance, feeling easily overwhelmed by things:

0	1	2	3	4
NONE	MILD	MODERATE	SEVERE	VERY SEVERE

23. Being unable to hear anything (complete deafness) for periods of time:

0	1	2	3	4
NONE	MILD	MODERATE	SEVERE	VERY SEVERE

24. Completely losing your voice for more than a minute:

0	1	2	3	4
NONE	MILD	MODERATE	SEVERE	VERY SEVERE

25. Difficulty swallowing due to a lump in throat:

0	1	2	3	4
NONE	MILD	MODERATE	SEVERE	VERY SEVERE

26. Seeing only in black and white:

0	1	2	3	4
NONE	MILD	MODERATE	SEVERE	VERY SEVERE

27. Complete loss of feeling in both arms:

0	1	2	3	4
NONE	MILD	MODERATE	SEVERE	VERY SEVERE

PCL-5

<u>Instructions</u>: **Below is a list of problems that people sometimes have in response to a very stressful experience. Please read each problem carefully and then circle one of the numbers to the right to indicate how much you have been bothered by that problem <u>in the past month</u>.**

In the past month, how much were you bothered by:	***Not at all***	***A little bit***	***Moderately***	***Quite a bit***	***Extremely***
**1. Repeated, disturbing, and unwanted memories of the stressful experience?**	**0**	**1**	**2**	**3**	**4**
**2. Repeated, disturbing dreams of the stressful experience?**	**0**	**1**	**2**	**3**	**4**
**3. Suddenly feeling or acting as if the stressful experience were actually happening again *(as if you were actually back there reliving it)*?**	**0**	**1**	**2**	**3**	**4**
**4. Feeling very upset when something reminded you of the stressful experience?**	**0**	**1**	**2**	**3**	**4**
**5. Having strong physical reactions when something reminded you of the stressful experience *(for example, heart pounding, trouble breathing, sweating)*?**	**0**	**1**	**2**	**3**	**4**
**6. Avoiding memories, thoughts, or feelings related to the stressful experience?**	**0**	**1**	**2**	**3**	**4**
**7. Avoiding external reminders of the stressful experience *(for example, people, places, conversations, activities, objects, or situations)*?**	**0**	**1**	**2**	**3**	**4**
**8. Trouble remembering important parts of the stressful experience?**	**0**	**1**	**2**	**3**	**4**
**9. Having strong negative beliefs about yourself, other people, or the world *(for example, having thoughts such as: I am bad, there is something seriously wrong with me, no one can be trusted, the world is completely dangerous)*?**	**0**	**1**	**2**	**3**	**4**
**10. Blaming yourself or someone else for the stressful experience or what happened after it?**	**0**	**1**	**2**	**3**	**4**
**11. Having strong negative feelings such as fear, horror, anger, guilt, or shame?**	**0**	**1**	**2**	**3**	**4**
**12. Loss of interest in activities that you used to enjoy?**	**0**	**1**	**2**	**3**	**4**
**13. Feeling distant or cut off from other people?**	**0**	**1**	**2**	**3**	**4**
**14. Trouble experiencing positive feelings *(for example, being unable to feel happiness or have loving feelings for people close to you)*?**	**0**	**1**	**2**	**3**	**4**
**15. Irritable behavior, angry outbursts, or acting aggressively?**	**0**	**1**	**2**	**3**	**4**
**16. Taking too many risks or doing things that could cause you harm?**	**0**	**1**	**2**	**3**	**4**
**17. Being “superalert” or watchful or on guard?**	**0**	**1**	**2**	**3**	**4**
**18. Feeling jumpy or easily startled?**	**0**	**1**	**2**	**3**	**4**
**19. Having difficulty concentrating?**	**0**	**1**	**2**	**3**	**4**
**20. Trouble falling or staying asleep?**	**0**	**1**	**2**	**3**	**4**

Severity Measure for Depression—Adult*

*Adapted from the Patient Health Questionnaire–9 (PHQ-9)

Name:_________________________________ **Age:** ______ **Sex: Male** ❑ **Female** ❑ **Date:**________________

Instructions: Over the **last 7 days**, how often have you been bothered by any of the following problems? (Use "✓" to indicate your answer)

						Clinician Use
						**Item score**
		**Not at all**	**Several days**	**More than half the days**	**Nearly every day**	
1.	Little interest or pleasure in doing things	0	1	2	3	
2.	Feeling down, depressed, or hopeless	0	1	2	3	
3.	Trouble falling or staying asleep, or sleeping too much	0	1	2	3	
4.	Feeling tired or having little energy	0	1	2	3	
5.	Poor appetite or overeating	0	1	2	3	
6.	Feeling bad about yourself—or that you are a failure or have let yourself or your family down	0	1	2	3	
7.	Trouble concentrating on things, such as reading the newspaper or watching television	0	1	2	3	
8.	Moving or speaking so slowly that other people could have noticed? Or the opposite—being so fidgety or restless that you have been moving around a lot more than usual	0	1	2	3	
9.	Thoughts that you would be better off dead or of hurting yourself in some way	0	1	2	3	
					**Total/Partial Raw Score:**	
					**Prorated Total Raw Score: (if 1-2 items left unanswered)**	

Adapted from Patient Health Questionnaire—9 (PHQ-9) for research and evaluation purposes.

Instructions to Clinicians

The Severity Measure for Depression—Adult (adapted from the Patient Health Questionnaire–9 [PHQ-9]) is a self-rated 9-item measure that assesses the severity of depressive symptoms in individuals age 18 and older. The measure is completed by the individual prior to a visit with the clinician. Each item asks the individual to rate the severity of his/her depression **during the last 7 days.**

Scoring and Interpretation

Each item on the measure is rated on a 4-point scale (0=Not at all; 1=Several days; 2=More than half the days; and 3=Nearly every day). The total score can range from 0 to 27, with higher scores indicating greater severity of depression. The clinician is asked to review the score of each item on the measure during the clinical interview and indicate the raw score for each item in the section provided for "Clinician Use." The raw scores on the 9 items should be summed to obtain a total raw score and should be interpreted using the Interpretation Table for the PHQ-9 below:

Interpretation Table for the PHQ-9

Levels of depressive symptoms severity	PHQ-9 Score
None	0-4
Mild depression	5-9
Moderate depression	10-14
Moderately severe depression	15-19
Severe depression	20-27

Note: If 3 or more items are left unanswered, the total score on the measure should not be calculated. Therefore, the individual should be encouraged to complete all of the items on the measure. If 1 or 2 items are left unanswered, you are asked to calculate a prorated score. The prorated score is calculated by summing the scores of the items that were answered to get a partial raw score. Multiply the partial raw score by the total number of items on the PHQ-9 (i.e., 9) and divide the value by the number of items that were actually answered (i.e., 7 or 8). The formula to prorate the partial raw score to Total Raw Score is:

$$\frac{\text{(Raw sum x 9)}}{\text{Number of items that were actually answered}}$$

If the result is a fraction, round to the nearest whole number.

Frequency of Use

To track changes in the severity of the individual's depression over time, the measure may be completed at regular intervals as clinically indicated, depending on the stability of the individual's symptoms and treatment status. Consistently high scores on a particular domain may indicate significant and problematic areas for the individual that might warrant further assessment, treatment, and follow-up. Your clinical judgment should guide your decision.
