# Supplementary material for: Traumatic Brain Injury Intensive Evaluation and Treatment Program: Protocol for a Partnered Evaluation Initiative Mixed Methods Study
Source: JMIR Res Protoc. 2023 May 9;12:e44776. doi: 10.2196/44776 (PMC10206625; doi:10.2196/44776)
Supplement: Multimedia Appendix 14 [file resprot_v12i1e44776_app14.pdf]

Appendix 14. Gantt Chart of Project Activities Across the 3-Year Project

ACTIVITY	YEAR 1				YEAR 2				YEAR 3			
	Q1[d]	Q2[e]	Q3[f]	Q4[g]	Q1	Q2	Q3	Q4	Q1	Q2	Q3	Q4
**Kick-off**	■											
Deliverable #1 (Yr. 1; Q1): MOU[a] of Operations Driven Deliverables												
**Aim 1** Recruitment	■	■	■									
**Aim 1** Data Collection		■	■	■	■							
**Aim 1** Transcription			■	■	■	■						
**Aim 1** Data Analyses			■	■	■	■	■					
Deliverable #2 (Yr. 2: Q3): IETP[b] Qualitative Results Report												
**Aim 2** Data Collection	■	■	■	■								
**Aim 2** Data Analysis				■	■	■	■					
Deliverable #3 (Yr. 2: Q3): IETP Quantitative Results Report												
**Aim 3** Develop Final Report	■	■	■	■	■	■	■	■	■			
**Aim 3** Present and Consult									■	■	■	■
Deliverable #4 (Yr. 3: Q1-Q4): Implementation Content Final Report and Presentation to Stakeholders<br>Deliverable #5 (Yr. 3: Q4): Final Report to PM&R[c]												

[a]MOU: Memorandum of Understanding.
[b]IETP: Intensive Evaluation and Treatment Program.
[c]PM&R: Physical Medicine and Rehabilitation.
[d]Q1: Quarter 1.

[e]Q2: Quarter 2.
[f]Q3: Quarter 3.
[g]Q4: Quarter 4.
